# Adaptive recognition network for few-shot plant diseases and pests based on homeostatic neuromodulation and meta-plasticity

**DOI:** 10.3389/fpls.2026.1796835

**Published:** 2026-04-29

**Authors:** Xiaoli Zhang, Yu Zhao, Kun Liang, Yanan Zhang

**Affiliations:** College of Artificial Intelligence, Tianjin University of Science and Technology, Tianjin, China

**Keywords:** BCM theory, few-shot learning, meta-plasticity, neuromodulation, plant disease and pest recognition

## Abstract

**Introduction:**

Static networks often exhibit limited generalization on few-shot data, particularly given the scarce samples and unstructured background noise inherent to precision agriculture. To address these limitations, an adaptive recognition network for few-shot plant diseases and pests based on homeostatic neuromodulation and meta-plasticity (HNeuroNet) is proposed.

**Methods:**

This framework incorporates dynamic plasticity inspired by biological systems to mitigate the data dependency paradox. First, a Neuro Modulatory Generator (NMG) is constructed utilizing a hypernetwork architecture. Simulating neurotransmitter gating mechanisms, affine transformation parameters are dynamically generated for feature channels based on support set samples. Consequently, instantaneous weight reconstruction is enabled without expensive gradient fine-tuning, thereby overcoming structural rigidity and catastrophic forgetting during rapid adaptation. Second, a Homeostatic Suppression Mechanism (HSM) integrating visual perception is introduced. Leveraging Bienenstock-Cooper-Munro (BCM) theory, an adaptive activation function is employed to regulate neuron thresholds based on historical feature map statistics. High-frequency noise from complex environments is suppressed, significantly enhancing feature extraction and target saliency in low signal-to-noise ratios. Finally, an end-to-end Dynamic Meta-Plasticity (DMP) strategy is implemented. By coupling parameter generation and threshold regulation within a bi-level optimization framework, biological homeostatic adaptation is simulated to adjust perception strategies. Context-dependent feature interaction patterns are established to secure robust discriminative boundaries under extreme few-shot conditions.

**Results:**

Experimental results demonstrate that HNeuroNet significantly outperforms state-of-the-art methods on IP102, PlantDoc, and Mini-ImageNet. Notably, 5-way 1-shot accuracy on the PlantDoc dataset surpasses the second-best baseline by 4.33%. Furthermore, a 1-shot accuracy of 71.36% is achieved on the cross-domain Mini-ImageNet task.

**Discussion:**

These results confirm the potential of bio-inspired computing in addressing data scarcity.

## 1 Introduction

Under the dual pressures of global climate change and population growth, maintaining food security and the stability of agricultural ecosystems has emerged as a critical issue demanding immediate resolution. Outbreaks of crop diseases and pests not only directly precipitate sharp declines in the quantity and quality of agricultural products but also inflict irreversible, sustained damage on regional ecological functions by disrupting biodiversity (Zhou et al., 2023). To effectively contain these biological threats, genetic control strategies (Ge et al., 2025) targeting specific destructive pests and entomological monitoring of vectors for virus transmission, such as African swine fever (Dhollander et al., 2025), have become key defensive lines in plant protection. However, existing pest and disease detection frameworks have long relied on traditional biochemical and molecular biological methods, including high-precision molecular detection techniques (Jhaiaun et al., 2025), real-time polymerase chain reaction (PCR) analysis based on environmental sampling (Loesing et al., 2025), and electronic nose technologies utilizing metal oxide semiconductor sensors (Zheng and Zhang, 2022). Although these methods demonstrate high diagnostic accuracy in controlled laboratory environments and have even been extended to biological monitoring of industrial pollution (Skaldina et al., 2023), their prohibitive detection costs, complex sample pre-processing workflows, and severe time lags fail to meet the urgent demands of modern precision agriculture for large-scale, real-time field monitoring. Consequently, breaking through the limitations of traditional biochemical detection to construct intelligent visual perception systems capable of adapting to unstructured field environments and providing rapid response capabilities has become a critical bottleneck that urgently needs to be overcome in the field of agricultural information technology.

Beyond visual perception tasks, the broader integration of Artificial Intelligence (AI) and the Internet of Things (IoT) has driven breakthrough advancements across diverse domains of precision agriculture. Recent notable studies have successfully deployed intelligent systems to address dynamic environmental factors. For instance, Khan et al. (2022a) proposed an IoT-assisted architecture for real-time soil fertility mapping, employing machine learning models to dynamically recommend fertilizers based on specific crop and soil contexts. In the domain of soil remediation, Khan et al. Khan et al. (2022b) developed an ensembled Long Short-Term Memory (LSTM) framework for context-aware evapotranspiration (ETs) estimation, which significantly improves the effective leaching processes required for saline soil reclamation. Furthermore, recent advancements in agricultural water management have seen the implementation of novel optimization algorithms to calibrate Reference Evapotranspiration (ETo) models, thereby achieving highly accurate precision irrigation Bashir et al (2023). Expanding upon these systems, advanced deep learning architectures are increasingly utilized for complex, real-time crop monitoring, such as cereal plant head detection and disease diagnosis Sanaeifar et al. (2023); Sharma et al. (2022). Simultaneously, enhanced IoT architectures utilizing rapid-adaptation algorithms have significantly improved Quality-of-Service (QoS) and energy harvesting for smart agriculture sensors in highly unstructured environments Singh et al. (2024). These advancements suggest that context-aware and dynamic optimization strategies are increasingly fundamental for addressing complex, non-stationary agricultural challenges, thereby providing a strong methodological inspiration for the adaptive recognition framework proposed in this study. Furthermore, the rise of Generative AI (Waikel et al., 2024) and unsupervised learning strategies (Liu et al., 2023) has provided new perspectives for solving problems related to automated modeling of biological features and learning with a lack of annotated data. The application of Automated Machine Learning (AutoML) has further lowered the deployment barrier for high-performance models in specific biomedical scenarios (Jacoba et al., 2023) (Cao et al., 2025), demonstrating potential that surpasses human experts in ultrasound and imaging diagnostics (Nakayama et al., 2023) (Ragnarsdottir et al., 2024).

However, despite the excellent performance of the aforementioned technologies in controlled environments such as laboratories, migrating them directly to unstructured open agricultural environments still faces a severe reality gap. Existing insect infection models (Banfi et al., 2024) (Watson et al., 2025) and behavioral analysis studies indicate that biological entities in natural environments exhibit high dynamism and unpredictability. Current mainstream static deep learning models rely heavily on large-scale, independent and identically distributed training data, lacking the dynamic adaptability required for field environments. Specifically, three core issues remain to be addressed. First, samples of emerging diseases are extremely scarce, making it difficult for data-hungry models to converge. Second, low signal-to-noise ratios caused by field illumination and complex textures make feature extractors based on static weights highly prone to generating false positives. Third, existing models lack homeostatic regulation mechanisms similar to biological nervous systems, rendering them unable to achieve instantaneous adaptation to new tasks without catastrophic forgetting. Therefore, constructing perception systems capable of dynamic plasticity and homeostatic noise resistance is key to breaking through the current bottlenecks in agricultural intelligent perception.

To address the aforementioned issues, we integrates computational neuroscience to propose an Adaptive Recognition Network for Few-shot Plant Diseases and Pests based on Homeostatic Neuromodulation and Meta-plasticity (HNeuroNet). The primary contributions are summarized as follows:

- A Neuro Modulatory Generator (NMG) based on a hypernetwork is proposed to resolve the structural rigidity dilemma in rapid few-shot adaptation. By simulating the gating mechanisms of biological neurotransmitters, this module dynamically generates task-specific channel-wise affine transformation parameters based on extremely scarce support set samples. This mechanism achieves instantaneous weight reconstruction under zero-gradient fine-tuning conditions, effectively overcoming the structural rigidity and catastrophic forgetting challenges inherent in traditional meta-learning when adapting to new categories.
- Homeostatic Suppression Mechanism (HSM) integrating BCM theory is designed to overcome feature extraction bottlenecks in low signal-to-noise ratio field environments. An adaptive activation function with sliding threshold characteristics is introduced to dynamically regulate neuron response thresholds according to the historical statistical distribution of feature maps. Functioning as an adaptive high-pass filter, this mechanism automatically suppresses high-frequency background noise caused by uncontrolled illumination or complex textures, significantly enhancing feature purity and target saliency in unstructured environments.
- An end-to-end Dynamic Meta-Plasticity (DMP) strategy is constructed to establish a context-dependent adaptive recognition mode. Parameter generation and threshold regulation are coupled within a bi-level optimization system, simulating the environmental homeostatic adaptation process of biological nervous systems to dynamically adjust perception strategies. This strategy establishes robust discriminative boundaries in the feature space under conditions of extreme data scarcity, not only improving cross-domain generalization capabilities but also providing a new theoretical perspective for the application of bio-inspired computing in smart agriculture.
- Experiments were conducted on a composite dataset comprising large-scale pest recognition (IP102), high-noise plant disease detection (PlantDoc), and a general few-shot benchmark (Mini-ImageNet). These experiments aim to verify the homeostatic suppression effectiveness of HNeuroNet against unstructured field noise and its cross-domain generalization potential under extreme data scarcity.

The remainder of this paper is organized as follows: Section 2 introduces related work, including plant disease and pest recognition methods. Section 3 describes the proposed method in detail. Section 4 presents quantitative and visualization analyses comparing the proposed method with other few-shot learning approaches to validate its classification performance on few-sample datasets. Section 5 discusses hyperparameter settings and efficiency. Finally, Section 6 provides the conclusion and future directions.

## 2 Related work

### 2.1 Deep learning architectures and applications in plant disease and pest recognition

Current paradigms for identifying plant diseases and pests are predominantly anchored in deep Convolutional Neural Networks (CNNs) and their variants. By training static architectures on extensive annotated datasets, these approaches leverage deep convolutional kernels to automatically extract visual features ranging from low-level textures to high-level semantics (Sajitha et al., 2024). This end-to-end learning paradigm has progressively superseded traditional manual feature engineering, establishing itself as the standard solution for multi-category classification and object detection tasks within precision agriculture monitoring, with the objective of achieving high-precision automated diagnosis.

To address the intricacies of agricultural scenarios, researchers have made significant strides in optimizing network architectures and enhancing feature representation. For instance, Naqi et al. Naqi et al. (2025) demonstrated the efficacy of deep transfer learning approaches in accurately detecting apple leaf diseases, while Khan et al. Khan et al. (2018) and Khan et al. Khan et al. (2020) proposed robust hierarchical frameworks leveraging deep feature fusion and correlation coefficients to significantly enhance fruit crop disease classification. Furthermore, high-precision morphological analysis has been advanced by Kolivand et al. Kolivand et al. (2019) through novel leaf venation detection techniques, and Mukhtar et al. Mukhtar et al. (2021) expanded macroscopic monitoring capabilities utilizing semi-supervised semantic segmentation for UAV-based plant counting. Furthermore, to optimize network architectures for complex agricultural scenarios, recent studies have made significant contributions to model deployment and holistic environmental analysis. For instance, Vishnoi et al. Vishnoi et al. (2023) developed an efficient 3-layer CNN for apple plant disease detection, demonstrating that streamlined architectures can achieve high diagnostic accuracy while reducing computational complexity. In the broader context of agricultural modeling, Parashar et al. Parashar et al. (2024) provided a comprehensive integrated analysis of crop yield prediction models, highlighting the critical importance of incorporating multifactorial environmental data to build robust predictive systems. These works provide excellent methods for efficient architectural design and comprehensive environmental factor integration. Building upon these insights, our proposed HNeuroNet focuses on a complementary challenge: extreme data scarcity and unstructured visual noise. While the aforementioned studies utilize efficient static network designs and multi-variable integration to enhance model robustness, HNeuroNet introduces a bio-inspired dynamic adaptation mechanism.

Despite these advancements, the aforementioned methods generally suffer from severe data dependency, with performance highly contingent upon massive volumes of pre-defined training samples. Moreover, traditional convolutional structures lack adaptive suppression capabilities against unstructured background noise, resulting in insufficient feature extraction purity within low signal-to-noise ratio environments. This limitation renders them inadequate for the robust perception requirements of complex open scenarios addressed in this study. The performance may degrade under extreme sample scarcity. HNeuroNet provides a complementary solution to this limitation. Unlike data-intensive methodologies, our framework achieves instantaneous dynamic plasticity, thereby securing robust performance even in 1-shot scenarios without extensive offline pre-training.

### 2.2 Noise-robust and adaptive learning strategies for few-shot scenarios

To address the challenges of sample scarcity and environmental non-stationarity prevalent in open agricultural scenarios, robust learning strategies aim to circumvent the reliance of traditional supervised learning on large-scale balanced data. By leveraging meta-learning, transfer learning, and multi-feature fusion, these approaches endow models with adaptive capabilities to handle long-tailed distributions and complex interferences. Mainstream research focuses on mining invariant features from limited samples, utilizing heterogeneous information complementarity or rapid parameter adaptation mechanisms to extend the generalization boundaries and noise resistance of models in dynamic environments.

In the specific domain of agricultural vision, recent studies have increasingly explored few-shot learning paradigms to mitigate the reliance on large-scale annotated datasets for plant disease recognition Li et al. (2024). For instance, several application-specific approaches have adapted prototypical networks architectures to identify rare leaf diseases by learning generalized distance metrics in a projected feature space (Fu et al., 2022). Other domain-specific solutions have incorporated cross-attention mechanisms to align query and support features under limited sample conditions, aiming to improve target localization on diseased crop leaves (Pourpanah et al., 2022). While these prior attempts effectively reduce data requirements, they predominantly rely on static metric spaces and context-independent feature extraction, lacking the dynamic plasticity necessary for highly unstructured field environments. Regarding rapid adaptation mechanisms, Dong (Dong, 2025) proposed a meta-learning strategy combined with multi-armed bandits, significantly accelerating convergence speeds in few-shot scenarios. To enhance feature robustness, multi-scale and multi-modal fusion have emerged as mainstream approaches: Dai et al (Dai et al., 2024). and Ali et al (Ali et al., 2024). strengthened the capture of disease details through the integration of multi-level depth information and heterogeneous features, respectively. Furthermore, Nandhini et al (Nandhini and Brindha, 2024). proposed a visual regenerative fusion network, employing generative adversarial concepts to repair impaired features. Addressing unstructured field noise, Chodey et al (Chodey and Noorullah Shariff, 2023). introduced fuzzy C-means segmentation and texture feature extraction to counteract background interference. P. V. et al (V. et al., 2024). utilized an Exponential Moving Average (EMA) fusion strategy to smooth prediction fluctuations, while Asadi-Aghbolaghi et al (Asadi-Aghbolaghi et al., 2024). and Omara et al (Omara et al., 2023). validated the effectiveness of such generalized models in multi-center and actual 183 field deployments.

Although the aforementioned strategies have achieved progress in specific tasks, they continue to face severe challenges. Existing fusion and adaptation mechanisms are predominantly based on static or pre-defined rules, lacking the homeostatic regulation capabilities based on environmental feedback found in biological neural systems. This absence of dynamic plasticity renders existing models incapable of establishing robust discriminative boundaries when identifying complex agricultural scenarios characterized by the coupling of extreme few-shot conditions and strong background noise. In contrast to these existing application-specific solutions, the proposed HNeuroNet introduces a fundamentally bio-inspired mechanism. This suggests that the model can dynamically adapt its perception strategy to emerging pests while simultaneously maintaining robust discriminative boundaries against complex agricultural background noise, thereby offering a highly resilient solution for real-world deployment.

### 2.3 Research motivation

(1) Traditional deep models frequently succumb to structural rigidity and catastrophic forgetting when adapting to new categories with extremely scarce samples. Current mainstream methods for plant disease and pest recognition predominantly rely on static convolutional neural networks (e.g., ResNet, YOLO series). These data-hungry models typically require massive quantities of annotated samples to achieve convergence. However, the distribution of insects and diseases in real-world agricultural ecosystems exhibits significant long-tailed characteristics, where samples of emerging diseases or invasive species are exceedingly rare. Although some studies have introduced meta-learning or few-shot adaptation strategies, these approaches generally depend on gradient-based fine-tuning processes. This update method is not only computationally expensive but also prone to disrupting the feature distributions learned by the model for established categories, resulting in catastrophic forgetting. In contrast, biological nervous systems can achieve instantaneous reconstruction of synaptic weights through neuromodulation. This dynamic parameter generation mechanism, which requires no extensive iteration, is precisely what existing computational models urgently need to resolve the conflict between rapid adaptation and memory retention.

(2) Unstructured field noise severely interferes with feature extraction, causing static models to frequently generate false detections under low signal-to-noise ratio conditions. Unlike clear images obtained in laboratory settings, disease and pest data collected in the field are replete with complex illumination changes, chaotic leaf vein textures, and soil background interference. Existing detection networks typically employ multi-scale feature fusion or attention mechanisms to enhance feature expression; however, their underlying activation functions (e.g., ReLU) are often static and lack the capacity for adaptive regulation based on the statistical characteristics of input signals. This implies that models cannot automatically filter ubiquitous high-frequency background texture noise via sliding threshold mechanisms (such as BCM theory) in the manner of the biological visual cortex. Consequently, when processing highly camouflaged or minute early-stage lesions, existing models are often overly sensitive to background noise, leading to degraded feature extraction purity and a high volume of false positive predictions, thereby failing to meet the requirements of precision agriculture for real-time, high-reliability detection.

(3) Existing vision systems lack the dynamic plasticity required for environmental adaptation, making it difficult to construct robust discriminative boundaries for extreme distributions. Current intelligent plant protection systems mostly adopt a static mode of training followed by deployment, lacking the capability to dynamically adjust perception strategies based on task context during the inference phase. Although some research has explored zero-shot learning or heterogeneous feature fusion, these methods typically decouple feature extraction from classification decisions and fail to establish a system-level homeostatic regulation mechanism similar to biological organisms. When enhancing extreme few-shot (e.g., 1-shot) tasks, this context-independent feature interaction mode cannot effectively measure the distance between new and prior knowledge, resulting in blurred discriminative boundaries. Therefore, there is an urgent need for an end-to-end bi-level optimization strategy that couples parameter generation with threshold regulation. By simulating the dynamic equilibrium process of biological nervous systems under varying environmental stimuli, a transformation from static pattern matching to dynamic situational awareness can be achieved.

## 3 Methods

**Figure 1:**
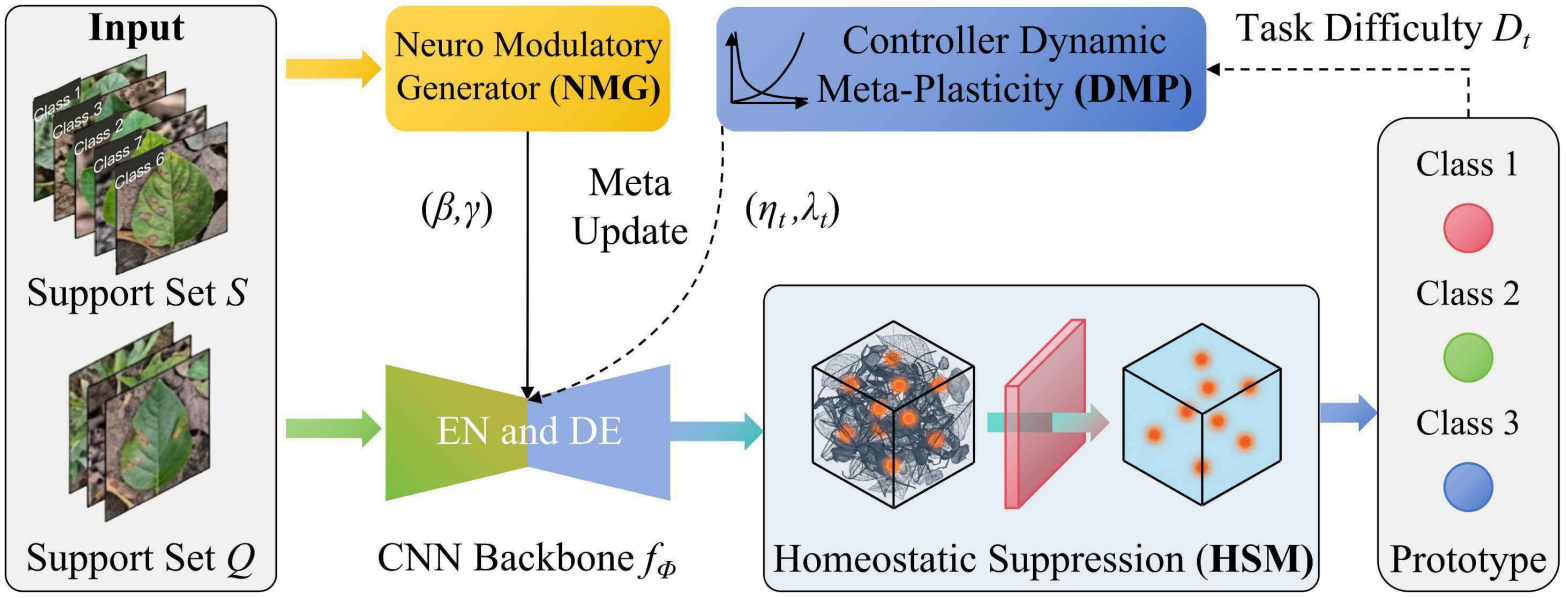
Overall architecture diagram of HNeuroNet.

As illustrated in **Figure 1**, the overall architecture of HNeuroNet follows a sequential workflow. First, the Neuro Modulatory Generator (NMG) extracts context from the support set constructed from plant disease and pest images to generate affine parameters (γ, β), which are used to instantaneously modulate the backbone features of the query set. Subsequently, the Homeostatic Suppression Mechanism (HSM) employs a dynamic sliding threshold ( ${\text{Θ}}_{t}$) to filter background noise from the modulated features, ensuring a high signal-to-noise ratio. Finally, the Dynamic Meta-Plasticity (DMP) strategy evaluates task difficulty $\left(D_{t}\right)$ to balance the plasticity $\left(\eta_{t}\right)$ and stability ( $\lambda_{t}$) of weight updates, thereby preventing catastrophic forgetting and enhancing the model’s classification accuracy.

### 3.1 Hypernetwork-based neuro modulatory generator

In traditional meta-learning or transfer learning frameworks, models typically necessitate iterative gradient fine-tuning to accommodate novel pest and disease categories. This paradigm is not only computationally.

intensive but also predisposed to disrupting feature distributions established for prior tasks, thereby inducing catastrophic forgetting. To resolve the conflict between structural rigidity and adaptive latency, we proposes the Hypernetwork-based Neuro Modulatory Generator (NMG). Designed to emulate the gating mechanisms of neuromodulators (such as dopamine) on synaptic plasticity, the NMG generates task-specific context regulatory parameters. These parameters facilitate the instantaneous reconstruction of feature extraction capabilities within the backbone network, endowing the model with rapid adaptation abilities for few-shot scenarios under zero-gradient update conditions.

**Figure 2:**
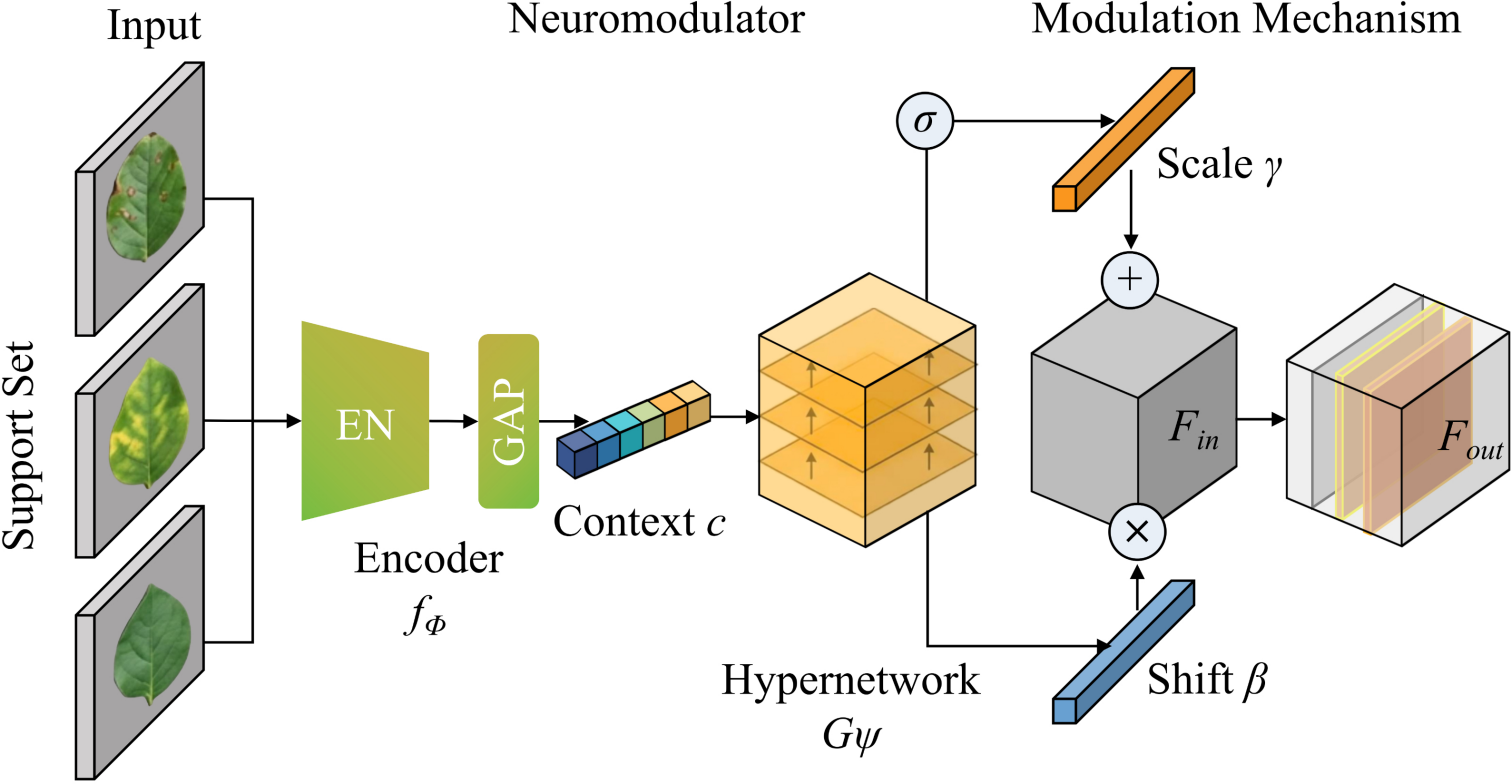
Overall architecture diagram of NMG.

The operational workflow of the NMG adheres to a biomimetic logic comprising extraction, generation, and modulation, as illustrated in **Figure 2**. Initially, the NMG accepts a limited number of samples from the Support Set, employing a shared lightweight feature encoder to extract feature representations reflecting the visual prototypes of the current disease category. These are statistically aggregated to construct a high-dimensional task context vector. Subsequently, this vector is fed into a hypernetwork architecture, which utilizes non-linear mapping to dynamically predict channel-wise affine transformation parameters (specifically, scaling and shifting factors) for distinct layers of the backbone network. Finally, these generated parameters are applied to the intermediate feature maps of the backbone via feature re-calibration, aligning the feature space with the new task without altering the core network weights.

First, the aggregation and representation of task context features. The primary objective of the NMG is to distill highly discriminative task representations from an exceedingly limited support set. In an *N*-way *K*-shot few-shot task setting, let the support set be denoted as $\;S={\left\{(x_{k},y_{k})\right\}}_{k=1}^{N\times K}$, where *x_k_*represents the input disease image and *y_k_*the corresponding label. To capture global semantic information, a pre-trained lightweight encoder *f_ϕ_*(·) first maps each image in the support set to the feature space, yielding local feature embeddings. To eliminate individual variations and extract category-specific prototypes, Global Average Pooling (GAP) and feature concatenation are employed to aggregate all support set features into a compact task context vector *c*, as defined in Equations 1, Equation 2 and Equation 3.

(1) $$z_{k}=f_{\phi }(x_{k}),\;x_{k}\in S_{n}$$

(2) $$p_{n}=\frac{1}{\left|S_{n}\right|}\sum_{x_{k}\in S_{n}}z_{k}$$

(3) $$c=\text{Concat}(p_{1},p_{2},\ldots ,p_{N})$$

Where $z_{k}\in {\mathbb{R}}^{D}$ denotes the single-sample feature, $S_{n}$ represents the sample subset for the *n*-th disease class, and $p_{n}$ is the prototype vector for the *n*-th class. The resulting $c\in {\mathbb{R}}^{N\cdot D}$ encodes the visual distribution information of all categories within the current task, serving as the seed signal for subsequent neuromodulator generation.

Second, the dynamic generation of affine parameters via the hypernetwork. Upon acquiring the task context vector *c*, the core function of the NMG is to transform it into specific parameters for regulating the backbone network. Inspired by hypernetworks, the NMG is designed as a parameter generation network $G_{\psi }(\cdot )$. Unlike approaches that directly predict large-scale convolutional kernel weights, the NMG predicts only channel-wise affine transformation parameters for feature modulation, drastically reducing computational complexity.

Specifically, for the *l*-th convolutional block in the backbone, a set of channel scaling factors $\gamma_{l}$ and translation factors $\beta_{l}$ must be generated. The NMG maps the high-dimensional context vector *c* to these parameter spaces via a Multi-Layer Perceptron (MLP). This process simulates the biological mechanism where neuromodulators are released based on environmental cues (context) to alter neuron response gains as Equations 4, Equation 5 and Equation 6.

(4) $$\left[\gamma_{l},\beta_{l}]=G_{\psi_{l}}(c\right)$$

(5) $$\gamma_{l}=\sigma (W_{\gamma_{l}}\cdot \text{ReLU}(W_{h}c+b_{h})+b_{\gamma })$$

(6) $$\beta_{l}=W_{\beta_{l}}\cdot \text{ReLU}(W_{h}c+b_{h})+b_{\beta }$$

Where $G_{\psi_{l}}$ denotes the generation sub-network for the *l*-th layer, and {W,b} are the learnable parameters of the NMG. The Sigmoid activation function σ(·) constrains the scaling factor to the (0,1) interval (or an adjusted rangebased on design requirements), while the translation factor $\beta_{l}$ remains unconstrained. The generated $\gamma_{l},\beta_{l}\in {\mathbb{R}}^{C_{l}}$ correspond strictly to the channel dimension $C_{l}$ of the *l*-th backbone layer.

Finally, channel-level feature re-calibration and modulation. The generated neuromodulatory parameters are applied to the feature extraction process of the backbone, enabling dynamic reconstruction of the feature flow. Let the input feature map of the *l*-th layer be $F_{in}^{l}\in {\mathbb{R}}^{H\times W\times C_{l}}$. Following the conventional convolution operation, a Feature Modulation operation is introduced. This operation does not alter the weights of the convolution kernels themselves but applies a specific linear transformation to each channel of the feature map. This mechanism resembles conditional batch normalization; however, the condition here is determined entirely by the support set *S*.

By applying the generated $\gamma_{l}$ and $\beta_{l}$ as slope and intercept, respectively, to the feature maps, the model enhances the activation of critical feature channels (distinguishing between two similar fungi) while suppressing irrelevant ones based on the current recognition task. It is shown in Equations 7 and Equation 8:

(7) $$F_{norm}^{l}=\frac{F_{in}^{l}-\mu_{l}}{{\left(\sigma_{l}\right)}^{2}+\in }$$

(8) $$F_{out}^{l}=\gamma_{l}⊙F_{norm}^{l}+\beta_{l}$$

Where $\mu_{l}$ and $\sigma_{l}$ represent the mean and standard deviation of the feature map, and ⊙ denotes channel-wise broadcasting multiplication. Through this approach, $F_{out}^{l}$ carries an inductive bias specific to the current few-shot task. This process achieves self-adaptation during the inference phase without requiriYng any gradient backpropagation fine-tuning, thereby effectively resolving the generalization performance bottleneck caused by fixed parameters in traditional models when confronting new diseases.

To explicitly delineate the biological grounding of the proposed architecture, we map the NMG to the action of diffuse neuromodulatory systems, such as the dopaminergic or serotonergic pathways in the mammalian brain. Biologically, these neuromodulators rapidly alter the gain and excitability of local neural circuits in response to novel environmental stimuli, achieving behavioral adaptation without waiting for the slow, iterative process of long-term synaptic consolidation. The proposed hypernetwork simulates this specific neural mechanism by projecting the global task context into local affine transformations. This suggests that the NMG effectively captures the functional benefit of instantaneous synaptic gain modulation, endowing the computational model with the ability to rapidly switch processing states and reconstruct feature channels when confronted with emerging, sample-scarce agricultural pests.

### 3.2 Homeostatic suppression mechanism integrating visual perception

Unstructured field environments characterized by illumination fluctuations, complex leaf vein textures, and soil backgrounds constitute high-frequency noise interference. This renders traditional convolutional networks, which rely on static activation functions (ReLU), incapable of distinguishing between disease features and background artifacts, thereby generating numerous false positives. To address this sensitivity to the signal-to-noise ratio, we proposes a Homeostatic Suppression Mechanism (HSM) integrating visual perception. This mechanism aims to dynamically filter environmental background noise by simulating the Bienenstock-Cooper-Munro (BCM) sliding threshold characteristics of the visual cortex, ensuring that the model responds solely to signals(diseases) that significantly deviate from the background statistical distribution.

**Figure 3:**
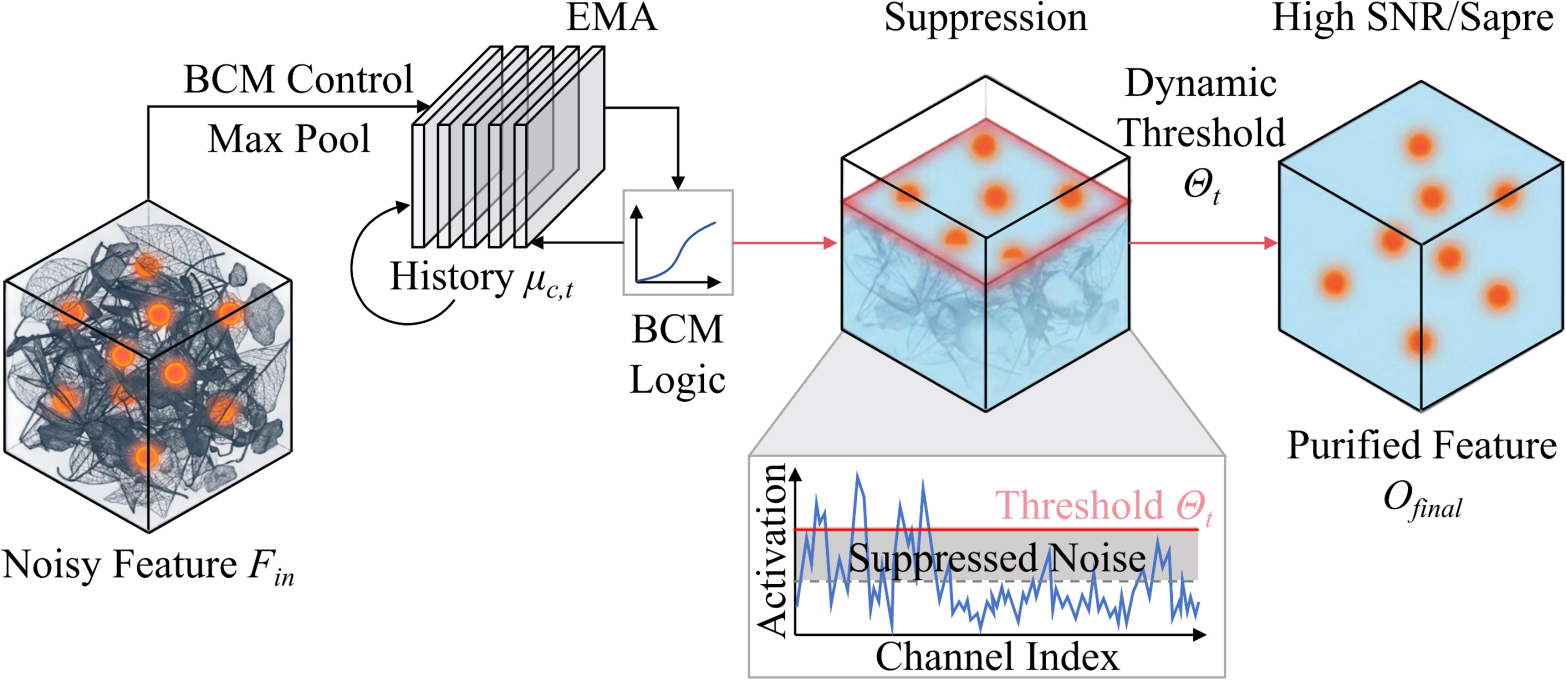
Overall architecture diagram of HSM.

The operational workflow of the HSM follows a closed-loop regulation logic of tracking, calculation, and gating, as illustrated in **Figure 3**. First, a neuron historical activity tracking queue is established, utilizing an exponential moving average strategy to update the statistical distribution prior of feature channels in the temporal dimension, thereby capturing the normative patterns of the environmental background. Next, a non-linear mapping function is constructed based on BCM homeostatic theory to calculate dynamic suppression thresholds for each channel according to historical activity, subjecting high-frequency background signals to higher activation thresholds. Finally, an adaptive sliding threshold activation function is designed to perform differential truncation on the feature responses output by the NMG module using the calculated dynamic thresholds, retaining only abnormal signals with high saliency to achieve high signal-to-noise ratio feature output.

Step 1: Temporal Tracking of Historical Activity. Background noise (e.g., wind-induced leaf movement, dappled light) typically manifests as continuous or high-frequency feature activation, whereas disease features appear as sudden, sparse signals. To distinguish between the two, a memory of the normative background must first be established. Following the preceding module (NMG), let the feature map of the *l*-th layer after neuromodulation be $F_{out}^{l}\in {\mathbb{R}}^{H\times W\times C_{l}}$. It is necessary to decouple channel-level activity intensity from the spatial dimension. To this end, Global Max Pooling is employed instead of average pooling to retain the most significant activation response $v_{c,t}^{l}$ within the current field of view, as the strongest response typically determines whether the channel is triggered.

To obtain stable background statistics, a momentum term *α* is introduced to perform Exponential Moving Average (EMA) in the temporal dimension. This effectively constructs a low-pass filter along the time axis, filtering out random perturbations in single-frame images and accumulating the long-term statistical characteristics $\mu_{t}^{l}$ of the environment. The process is shown in Equations 9 and Equation 10.

(9) $$v_{c,t}^{l}=\underset{i,j}{\max}(F_{out}^{l}(i,j,c))$$

(10) $$\mu_{c,t}^{l}=\alpha \cdot \mu_{c,t-1}^{l}+(1-\alpha )\cdot v_{c,t}^{l}$$

Where $\mu_{c,t}^{l}$ represents the Historical Average Activity of the *c*-th channel in the *l*-th layer at time *t*. This variable serves as a homeostatic memory unit, recording the prevalent activation level of the feature channel in the recent environment to provide a baseline for subsequent threshold calculations.

Step 2: Dynamic Threshold Generation Based on BCM Theory. According to BCM theory in neurobiology, the synaptic plasticity threshold of a neuron is not fixed but is a non-linear function of its historical activity. When a neuron (or feature channel) remains in a high activation state for an extended period (a channel detecting green leaves is consistently activated in the field), its threshold should automatically rise to prevent hypersensitivity. The threshold for low-frequency channels should decrease to maintain sensitivity.

We mathematically formulate this biological mechanism as the calculation process for the Dynamic Suppression Threshold $\theta_{t}^{l}$. We define the threshold generation function H(·) as a super-linear power function of historical activity. This non-linear design enhances the penalty for strong background noise, meaning the degree of suppression increases exponentially with background signal strength. Simultaneously, a learnable sensitivity coefficient *λ* is introduced to balance the suppression intensity, as shown in Equations 11 and Equation 12.

(11) $$\theta_{c,t}^{l}=H(\mu_{c,t}^{l})=\lambda \cdot {\left(\mu_{c,t}^{l}\right)}^{p}$$

(12) $${\text{Θ}}_{t}^{l}=\text{Expand}(\theta_{t}^{l})\in {\mathbb{R}}^{1\times 1\times C_{l}}$$

Where *p* is the BCM non-linear exponent (typically set to *p* = 2 to simulate energy relationships), and $\theta_{t}^{l}$ is the tensor resulting from expanding the channel thresholds to match the dimensions of the feature map. This step ensures that each feature channel possesses an independent gating standard, enabling the model to adaptively ignore redundant features that are ubiquitous in the current environment.

Step 3: Adaptive Sliding Threshold Activation and Feature Gating. The dynamic thresholds are applied to the feature maps to physically filter out background noise. Unlike the traditional ReLU activation function max (0*, x*), which hard-codes the threshold to 0, HSM employs a Shifted Rectified Linear Unit. This activation function subtracts the corresponding dynamic threshold $\theta_{t}^{l}$ from the input feature response. A neuron fires only when the current instantaneous activation intensity significantly exceeds the historical background level (*x > θ*); otherwise, the signal is forced to zero.

To further smooth the gradient flow and prevent the dead neuron problem, a minimal leakage slope is introduced in the suppression zone, and original features are fused via a residual connection to form the final output feature map $O_{final}^{l}$, as shown in Equation 13 and Equation 14.

(13) $$A_{act}^{l}=\text{max }(0,F_{out}^{l}-{\text{Θ}}_{t}^{l})$$

(14) $$O_{final}^{l}=A_{act}^{l}+\beta \cdot F_{out}^{l}$$

Where *A^lact^*is the purified feature after homeostatic suppression, and *β* is an extremely small residual coefficient. Through this mechanism, HSM effectively functions as an adaptive high-pass filter, dynamically stripping away low-frequency steady-state background information such as illumination and soil. This significantly enhances the signal-to-noise ratio (SNR) of disease and pest targets in the feature space, providing a robust discriminative basis for the subsequent classifier.

Furthermore, the connection to homeostatic neuromodulation is deeply rooted in the principle of homeostatic synaptic plasticity, whereby biological neurons dynamically adjust their activation thresholds to prevent runaway excitation or complete quiescence. In the mammalian visual cortex, the BCM theory mathematically postulates that the threshold for synaptic potentiation slides based on the neuron’s time-averaged activity. We computationalize this specific neural mechanism by utilizing the Exponential Moving Average (EMA) to track historical channel activity, thereby constructing a dynamic sliding threshold. The critical functional benefit of this homeostatic regulation is the maintenance of a stable representation landscape. It prevents the network from becoming hypersensitive to ubiquitous, high-frequency environmental noise, ensuring that the model selectively fires only in the presence of salient anomalies, such as disease lesions. This biologically-plausible background suppression is essential for maintaining feature purity in unstructured field environments.

### 3.3 End-to-end dynamic meta-plasticity strategy

Current few-shot recognition frameworks predominantly employ static pre-training and fine-tuning paradigms, lacking system-level mechanisms to dynamically adjust perception strategies based on task context during the inference phase. This deficiency prevents discriminative boundaries in the feature space from converging under extreme distributions (1-shot). Furthermore, isolated parameter generation and threshold suppression without a unified optimization objective are prone to training instability caused by local optima. To address these issues, we proposes the End-to-End Dynamic Meta-Plasticity (DMP) strategy. This strategy aims to construct an end-to-end bi-level optimization system that couples local neuromodulation with global homeostatic regulation, simulating the dynamic equilibrium process of biological nervous systems under varying environmental stimuli, thereby establishing robust and generalized discriminative boundaries.

**Figure 4:**
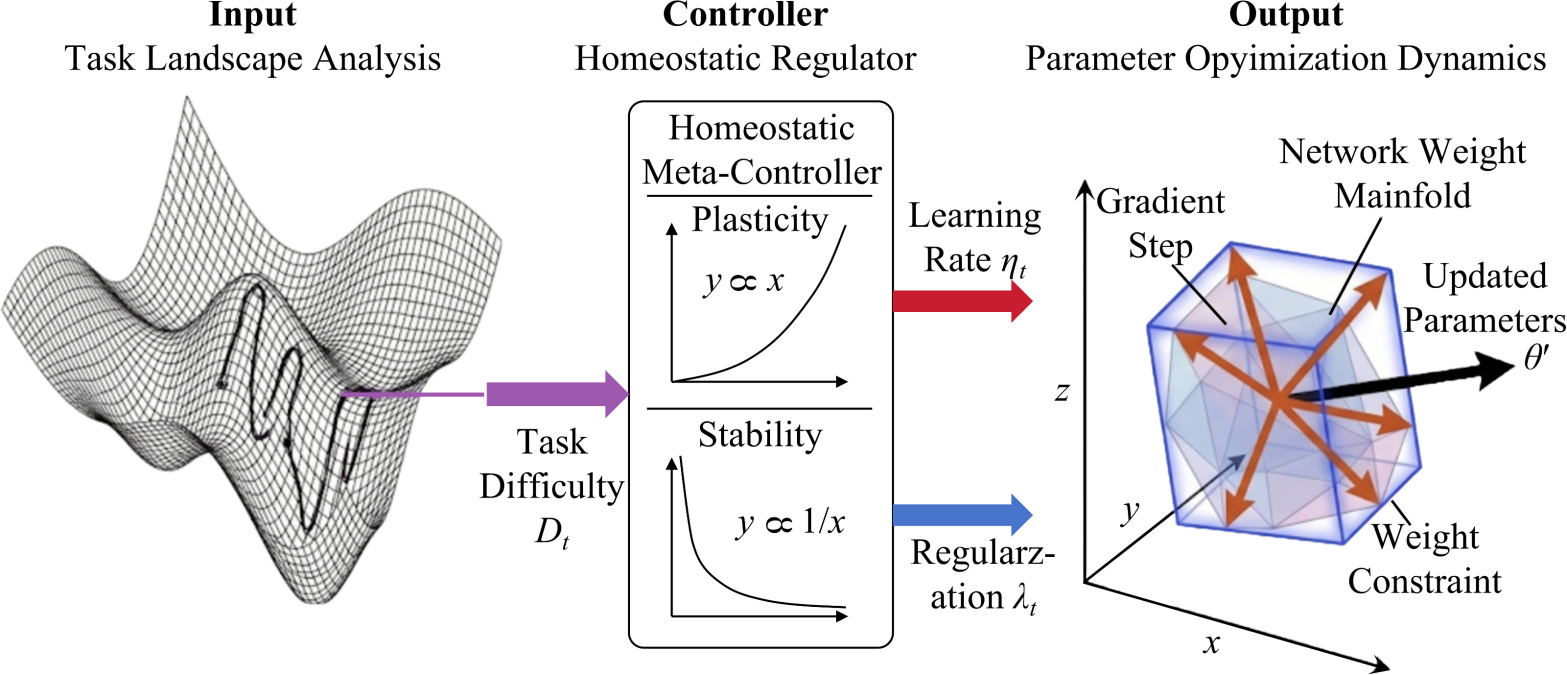
Overall architecture diagram of DMP.

The operational workflow of DMP adheres to a system-level optimization logic comprising coupling, metric measurement, and evolution, as illustrated in **Figure 4**. First, a context-dependent forward propagation path is established, embedding the affine parameters generated by the NMG and the homeostatic thresholds calculated by the HSM into the backbone network to form a dynamic feature extraction flow. Second, a prototype-based metric space is constructed; the cosine distance between the query set samples and support set prototypes is calculated using purified features subject to homeostatic suppression to define the meta-task loss function. Finally, a bi-level meta-optimization algorithm is implemented. Through an outer loop, hypernetwork parameters and homeostatic sensitivity coefficients are updated, enabling the model to learn how to adjust its own structure in response to the environment, thereby achieving adaptive inference without gradient fine-tuning when confronting new tasks.

(1) Context-Dependent Dynamic Forward Propagation. The objective of DMP is to seamlessly integrate the aforementioned NMG and HSM modules into each layer of the backbone network, constructing a feature extractor Φ capable of meta-plasticity. Unlike traditional convolutional networks *f*(*x*;*W*) where weights *W* are fixed, the effective weights in the DMP network are functions of the input support set *S*. For any input sample *x*, the feature extraction process becomes explicitly dependent on the task context *c*.

Let the composite transformation of the *l*-th layer be *T_l_*. This transformation first receives the neuromodulatory parameters ( $\gamma_{l},\;\beta_{l}$) generated by the NMG module $G_{\psi l}($*c*) to perform feature reconstruction, and subsequently utilizes the dynamic threshold $\Theta_{l}$calculated by the HSM module H(·) for homeostatic activation. Assuming the output of the previous *l* − 1 layers for *x* is *O_l_*_−1_, the dynamic propagation process for the *l*-th layer is as Equations 15, Equation 16 and Equation 17.

(15) $$\left[\gamma_{l},\beta_{l}]=G_{\psi_{l}}(c),\;{\text{Θ}}_{l}=H(\text{EMA}(O_{l-1})\right)$$

(16) $$F_{l}=\text{Conv}(O_{l-1};W_{l})⊙\gamma_{l}+\beta_{l}$$

(17) $$O_{l}=\text{ReLU}(F_{l}-{\text{Θ}}_{l})+\beta \cdot F_{l}$$

By stacking these transformations layer by layer, the finally obtained feature embedding z = Φ(x;W,ψ,c) not only contains visual information of the image but also fuses adaptive biases for the current task. In this formula, *W* represents the basic static weights of the backbone network, and *ψ* represents the hyperparameters of the NMG. This design ensures that the model retains general visual feature extraction capabilities while possessing instantaneous plasticity for specific disease and pest tasks.

(2) Prototype Metric Space and Meta-Task Loss Definition. Upon obtaining the dynamically modulated feature embeddings, the DMP strategy adopts a metric-based meta-learning mode to finalize classification decisions. We posit that the feature space after HSM homeostatic suppression possesses higher intra-class compactness and inter-class separability.

For the *n*-th disease class in the current task *T_i_*, the prototype center $P_{n}$is calculated using the mean of the support set sample features, as shown in Equation 18.

(18) $$P_{n}=\frac{1}{\left|S_{n}\right|}\sum_{x_{k}\in S_{n}}\text{Φ}(x_{k};W,\psi ,c)$$

For a sample *x_q_*in the Query Set, we calculate the cosine similarity between it and each category prototype *P_n_*, and generate a predicted probability distribution using the Softmax function. To further enhance the model’s focus on hard examples, we employ scaled cosine distance and introduce a temperature coefficient *τ*. As shown in Equations 19 and Equation 20.

(19) $$p(y=n\text{|}x_{q})=\frac{\text{exp }(\text{sim}(x_{q},P_{n})\cdot \tau )}{\sum_{j=1}^{N}\exp\;(\text{sim}(x_{q},P_{j})\cdot \tau )}$$

(20) $$L_{T_{i}}=-\sum_{(x_{q},y_{q})\in Q_{i}}\text{log }p(y_{q}|x_{q})$$

Where *Q_i_*represents the query set of task *T_i_*, and sim(·) denotes cosine similarity6. This loss function $L_{T_{i}}$ directly measures the generalization ability of the model on unseen samples under the influence of the current NMG parameters and HSM thresholds.

(3)Bi-Level Meta-Optimization and Parameter Evolution. To endow the model with genuine learning-to-learn capabilities, DMP employs an episodic bi-level optimization strategy. Unlike MAML, which requires updating backbone weights in the inner loop, this framework utilizes the NMG to achieve gradient-free rapid adaptation; consequently, the optimization focus is placed on the outer loop. The objective is to identify the optimal hyperparameter configuration such that the generated modulation parameters and calculated thresholds perform optimally across diverse tasks.

We denote the set of all learnable parameters as Λ={W,ψ,λ,α}, which includes backbone weights, hypernetwork parameters, BCM sensitivity, and momentum coefficients. The training process is conducted on a large number of sampled meta-task batches ${\left\{T_{i}\right\}}_{i=1}^{B}$. The optimization goal is to minimize the expected loss on the query sets across all tasks, as shown in Equation 21.

(21) $$\underset{\text{Λ}}{\min}J(\text{Λ})=E_{T_{i}∼P(T)}[L_{T_{i}}(Q_{i};\text{Φ}(S_{i},\text{Λ}))]$$

Parameter updates follow the Stochastic Gradient Descent (SGD) direction, calculating cumulative gradients via the backpropagation algorithm. Notably, the gradient flow traverses the prototype calculation steps, HSM threshold generation steps, and NMG parameter generation steps to directly update the underlying meta-parameters, as shown in Equation 22.

(22) $$\text{Λ}\leftarrow \text{Λ}-\eta \cdot \frac{1}{B}\sum_{i=1}^{B}{\text{∇}}_{\text{Λ}}L_{T_{i}}$$

This optimization process forces the NMG to learn to capture commonalities and differences between distinct diseases and pests, while simultaneously compelling the HSM to learn how to set optimal noise suppression thresholds based on statistical regularities. To endow the model with genuine learning-to-learn capabilities, DMP employs an episodic bi-level optimization strategy. However, the theoretical foundation of DMP differs fundamentally from standard meta-learning frameworks such as MAML. While MAML relies on explicit, computationally expensive gradient steps in the inner loop to update backbone weights for task adaptation, DMP achieves inner-loop adaptation implicitly via the zero-gradient parameter generation of the NMG.

By replacing explicit gradient updates with instantaneous forward-pass parameter modulation, DMP decouples the acquisition of task-specific plasticity from the updating of core network weights. This implicit adaptation mechanism may significantly reduce computational latency while effectively mitigating the catastrophic forgetting typically induced by iterative gradient fine-tuning. Consequently, the optimization focus is entirely shifted to the outer loop. The objective is to identify the optimal meta-parameter configuration such that the generated modulation parameters and calculated thresholds perform optimally across diverse unstructured tasks.

### 3.4 Loss function and optimization objective

To ensure HNeuroNet establishes robust discriminative boundaries under extreme few-shot conditions and to compel the HSM to effectively filter unstructured field background noise1, we construct a composite objective function comprising episodic classification loss and homeostatic sparse regularization. Within the bi-level optimization framework, this function synergistically guides the parameter evolution of the NMG and the dynamic reconstruction of the feature space.

First, a prototype-based metric learning paradigm is adopted for the classification task. For the query set feature embedding *z_q_*, which has undergone NMG modulation and HSM suppression, the cosine similarity with each disease prototype *P_n_*is calculated. To mitigate feature distribution variance and mine hard examples, a learnable temperature coefficient *τ* is introduced to scale the similarity. Consequently, the posterior probability distribution of a query sample belonging to the *n*-th class is defined as Equation 23.

(23) $$p(y=n\text{|}x_{q})=\frac{\text{exp}\;(\tau \cdot \text{cos}\;(z_{q},P_{n}))}{\sum_{j=1}^{N}\text{exp}\;(\tau \cdot \text{cos}\;(z_{q},P_{j}))}$$

Second, to prevent the dynamic thresholds of the HSM from degenerating to zero and to simulate the energy minimization coding principle of the biological visual cortex, a homeostatic sparse regularization term based on the *L*_1_ norm is introduced. This regularization term compels the model to elevate suppression thresholds to filter ubiquitous redundant background textures, ensuring that the final output feature map $O_{final}$possesses high sparsity and a high signal-to-noise ratio. In summary, the total optimization objective is to minimize the weighted sum of classification error and feature sparsity over the sampled meta-task distribution. By jointly updating the meta-parameter set Λ, the total loss is defined as Equation 24.

(24) $$J(\text{Λ})=E_{T_{i}}[-\frac{1}{\left|Q_{i}\right|}\sum_{x_{q}\in Q_{i}}\text{log }p(y_{q}\text{|}x_{q})+\eta \sum_{l=1}^{L}\frac{∥O_{final}^{l}∥}{C_{l}H_{l}W_{l}}]$$

Where *η* is a hyperparameter balancing classification accuracy and background suppression intensity2. This optimization objective prompts the model to internalize the homeostatic regulation strategy for ignoring environmental noise while learning to recognize specific diseases.

### 3.5 Novelty and incremental contributions

To explicitly delineate the incremental contributions of the proposed HNeuroNet over existing state-of-the-art approaches, we conceptually compare our framework across three critical dimensions: adaptation mechanism, noise suppression, and system-level optimization.

First, regarding the adaptation mechanism, unlike recent prompt-tuning methods or traditional meta-learning frameworks that rely on computationally expensive gradient fine-tuning for task adaptation, NMG introduces a zero-gradient, instantaneous weight reconstruction mechanism. This suggests a fundamental shift from iterative learning to dynamic parameter generation, significantly reducing adaptation latency and computational overhead.

Second, in terms of noise suppression, existing robust strategies typically utilize static feature fusion or fixed attention mechanisms. In contrast, HSM integrates BCM theory to establish an adaptive sliding threshold. This provides a biologically-plausible high-pass filter that dynamically adjusts to historical background statistics, offering superior suppression of unstructured field noise compared to static activation functions.

Finally, concerning system-level optimization, rather than employing context-independent feature extraction followed by metric classification, DMP strategy couples parameter generation and threshold regulation within an end-to-end bi-level optimization system. This simulates biological homeostatic equilibrium, which may indicate a more resilient methodology for securing robust discriminative boundaries under extreme data scarcity (1-shot scenarios) than conventional disjointed learning paradigms.

## 4 Experimental results and analysis

### 4.1 Datasets and comparative models

To comprehensively evaluate the performance of HNeuroNet across three core dimensions—few-shot adaptability, homeostatic noise regulation, and cross-domain generalization capabilities—we selected three public datasets for experimentation. These include two specialized agricultural datasets and one general few-shot learning benchmark.

- The IP102 dataset (Wu et al., 2019) contains over 75,000 images covering 102 fine-grained insect pest categories. Regarding the acquisition protocol, the dataset was constructed by systematically collecting images from major agricultural extension systems, field surveys, and targeted web crawling, resulting in complex field backgrounds such as crop occlusion and uncontrolled illumination. The class distribution exhibits a natural, highly imbalanced long-tailed characteristic; the number of samples per class varies significantly, ranging from fewer than 100 to over 5,000 images. Its categorical richness and environmental diversity make it an ideal benchmark for assessing the NMG module’s ability to generate adaptive parameters under extreme few-shot conditions and the HSM module’s capacity to suppress high-frequency noise in complex backgrounds.
- The PlantDoc dataset (Singh et al., 2020) consists of 2,598 images covering 27 disease categories across 13 plant species. For the acquisition protocol, all images were crawled from the internet and subsequently verified by agricultural domain experts, exhibiting highly unstructured characteristics, including cluttered backgrounds and multiple overlapping leaves. The class distribution is more restricted, with categories containing roughly 50 to 200 images each. Unlike the laboratory-controlled PlantVillage dataset, the high-noise nature of PlantDoc strictly validates the robustness of the proposed homeostatic suppression mechanism in stripping away background artifacts and enhancing lesion signal-to-noise ratios in real-world agricultural scenarios.
- The Mini-ImageNet (Vinyals et al., 2016) few-shot learning benchmark dataset contains 100 categories uniformly sampled from the ILSVRC-2012 ImageNet dataset. The acquisition protocol follows the standard benchmark sampling strategy, ensuring a strictly balanced class distribution of exactly 600 images per category, totaling 60,000 images. The dataset is typically divided into 64 classes for meta-training, 16 for meta-validation, and 20 for meta-testing. Introducing this dataset aims to perform a cross-domain evaluation, verifying whether the dynamic meta-plasticity established by the DMP strategy possesses theoretical universality beyond specific agricultural domains.
- To ensure a comprehensive and rigorous evaluation, the baseline models were selected based on strict inclusion criteria: they must represent the latest state-of-the-art advancements across distinct, modern architectural paradigms relevant to few-shot agricultural vision. Specifically, QSFormer and Hiller et al. were included as representative modern Transformer-based architectures to evaluate the efficacy of long-range dependency modeling. KLSANet and Askari et al. were selected as hybrid CNN-attention and multi-scale feature models to assess local semantic alignment and fine-grained texture capture. Finally, AMPL was included to represent the cutting-edge prompt-tuning paradigm. This diverse baseline selection ensures that the incremental contributions of the proposed bio-inspired framework are validated against a comprehensive spectrum of modern network architectures.
- AMPL (Wu et al., 2025) proposes an Adaptive Meta-Prompt Learner (AMPL). By dynamically generating visual prompts using image patch features combined with a token-aware enhancement module, it aims to address the insufficient adaptability of pre-trained models in diverse downstream few-shot tasks, representing the latest advancements in prompt tuning techniques for visual classification.
- Askari et al (Askari et al., 2025). constructed a feature extraction framework integrating learnable multi-scale embeddings and self-attention mechanisms. By performing weighted fusion of feature representations at different levels to capture global structures and local textures, this method primarily addresses the issue of singular feature representation caused by target scale variations in few-shot scenarios.
- QSFormer (Wang et al., 2023) deeply integrates few-shot learning with Transformer architectures, proposing the Unified Query-Support Transformer (QSFormer). It utilizes a global sample branch and a local patch branch to simultaneously model long-range dependencies and semantic associations between query and support sets, providing a strong baseline based on self-attention mechanisms.
- KLSANet (Sun et al., 2024) designed the Key Local Semantic Alignment Network (KLSANet) to address background noise interference. It precisely aligns critical local regions through hierarchical embedding and semantic pixel matching modules, and introduces a screening mechanism to mitigate the negative impact of non-semantic backgrounds on classification discrimination.
- Hiller et al (Hiller et al., 2022). revisited the generalization mechanism in few-shot classification, proposing the use of visual Transformers to establish category-independent local semantic correspondences. They validated the theoretical and practical value of self-supervised mask modeling strategies in enhancing cross-domain generalization capabilities.

### 4.2 Experimental environment and evaluation metrics

To validate the efficacy of the proposed HNeuroNet framework and ensure the fairness and reproducibility of the experimental results, all experiments were conducted within the computational environment detailed in **Table 1**. During the training phase, the network parameters were updated utilizing the Adam optimizer with an initial learning rate of 1 × 10^−3^. To mitigate overfitting, dropout with a rate of 0.3 was applied to the fully connected layers. The reported experimental results represent the average values derived from multiple independent trials. With the exception of the Mini-ImageNet dataset, where the default input image resolution of 84 × 84 was maintained, all other images were resized to a uniform dimension of 224 × 224 pixels.

**Table 1:** Experimental environment settings.

Environment	Configuration	Environment	Configuration
CPU	Intel(R) Xeon(R) Gold 6226R @ 2.90GHz	Framework	PyTorch 1.13.1, CUDA 11.7
GPU	NVIDIA RTX 4090	Learning Rate	1 × 10−3
OS	Ubuntu 20.04 LTS	Batch Size	32
Language	Python 3.8.10	Training Epochs	200

To provide a comprehensive and objective assessment of the effectiveness and robustness of HNeuroNet in few-shot plant disease and pest recognition tasks, this study employs multi-dimensional quantitative metrics. Addressing the stochastic sampling nature of meta-learning tasks, Average Classification Accuracy (*Acc*) is adopted as the primary metric. Assuming that within *E* randomly sampled episodes, the *i*-th episode contains a total of *Q_i_*query set samples with *M_i_*correctly classified instances, the *Acc* and its 95% confidence interval (*CI*) are defined as follows:


$$Acc=\frac{1}{E}\sum_{i=1}^{E}\frac{M_{i}}{Q_{i}}\times 100\%$$

$$CI=Acc\pm 1.96\;.\;\;\frac{\sigma }{\sqrt{E}}$$

Where *σ* represents the standard deviation of the accuracy, and the confidence interval serves to quantify the statistical stability of the model across different task samplings. To provide a comprehensive and objective assessment of the effectiveness and robustness of HNeuroNet, and to rigorously validate its generalizability across limited scenarios, this study employs a strict 5-fold cross-validation protocol. The datasets were partitioned into five mutually exclusive folds, with each fold iteratively serving as the meta-testing set. Addressing the stochastic sampling nature of meta-learning tasks, Average Classification Accuracy (Acc) is adopted as the primary metric. The final performance is reported as the Mean ± 95% Confidence Interval (Mean ± 95% CI) across all folds. This statistical representation effectively quantifies the model’s stability and variability, ensuring that the evaluated performance boundaries are robust against specific data partition biases.

Furthermore, given that background noise in field environments may induce fluctuations in false positive rates, accuracy alone is insufficient to fully capture model performance on hard examples. Consequently, the Macro-Average F1 Score (*F*1) is introduced to evaluate the equilibrium between precision and recall. This metric, calculated as the arithmetic mean of the harmonic means across all *N* categories, effectively verifies the contribution of the HSM in enhancing feature purity:


$$F1=\frac{1}{N}\sum_{c=1}^{N}\frac{2\times P_{c}\times R_{c}}{P_{c}+R_{c}}$$

Here, *P_c_*and *R_c_*denote the precision and recall, respectively, for the *c*-th disease category. A high F1 score indicates that the model maintains high sensitivity in capturing minute lesions while simultaneously suppressing background artifacts.

### 4.3 Analysis of few-shot pest and disease recognition effects

To evaluate the effectiveness of the proposed methods, comparative experiments were conducted across three datasets. As evidenced by the experimental results in **Table 2**, HNeuroNet achieved performance significantly superior to existing state-of-the-art (SOTA) methods on the IP102 dataset. Particularly in the most challenging 5-way 1-shot task, its accuracy reached 66.85%, exceeding the second-best model, AMPL, by approximately 4.1 percentage points. This advantage is primarily attributed to the dynamic reconstruction capability of the NMG; unlike the meta-prompt fine-tuning of AMPL or the multi-scale embeddings of Askari et al., the NMG can instantaneously generate affine parameters adapted to a single sample. This effectively overcomes the extreme long-tailed distribution issues inherent in insect species. Furthermore, regarding the complex field backgrounds of IP102, although KLSANet attempted to mitigate noise through local alignment, the substantial lead of HNeuroNet in Macro-F1 scores confirms the superiority of the HSM. By simulating the sliding threshold characteristics of biological vision, the HSM functions as an adaptive high-pass filter, peeling away environmental artifacts more robustly than Transformer-based methods (QSFormer). In summary, HNeuroNet achieves optimal feature discriminability under the dual constraints of data scarcity and environmental interference by simulating biological homeostatic plasticity.

**Table 2:** Comparison of few-shot classification performance on the IP102 dataset.

Method	5-way 1-shot		5-way 5-shot	
	Acc	F1	Acc	F1
Hiller et al.	54.12 ± 0.65	52.8	71.35 ± 0.54	70.1
QSFormer	58.45 ± 0.58	57.2	76.12 ± 0.49	75.4
KLSANet	60.82 ± 0.61	59.5	78.04 ± 0.52	77.1
Askari et al.	61.93 ± 0.55	60.8	79.25 ± 0.46	78.5
AMPL	62.74 ± 0.52	61.5	80.10 ± 0.43	79.2
**HNeuroNet**	**66.85 ± 0.48**	**65.9**	**83.42 ± 0.39**	**82.7**

Reported results are Acc(%) with 95% confidence intervals (CI) and F1 scores (%). Bold data indicates optimal results.

As shown in **Table 3**, HNeuroNet demonstrated overwhelming performance advantages on the PlantDoc dataset, which is characterized by extremely high unstructured noise. Unlike the results on the IP102 dataset, KLSANet surpassed AMPL and Askari et al. to rank second on this dataset. This is because KLSANet’s unique local semantic alignment mechanism can mitigate background interference to a certain extent. However, HNeuroNet still outperformed KLSANet by 4.33% in the 5-way 1-shot task. This proves that the proposed HSM is more thorough and robust than KLSANet’s alignment strategy when dealing with “hard” noise such as complex lighting and leaf overlap. Notably, the improvement in the F1 score for HNeuroNet was slightly higher than the improvement in accuracy. According to the definitions in the experimental setup, this implies that the model maintains high sensitivity while suppressing background artifacts. In the extreme scenario of 1-shot learning, which lacks prior information, HNeuroNet maintained an accuracy of over 62%, whereas the Transformer-based QSFormer achieved only 52.68%. This indicates that the parameter reconstruction capability provided by the NMG enables the model to rapidly adapt to new noise distributions via meta-learned homeostatic strategies without relying on massive samples for denoising training.

**Table 3:** Comparison of few-shot classification performance on the PlantDoc dataset.

Method	5-way 1-shot		5-way 5-shot	
	Acc	F1	Acc	F1
Hiller et al.	48.25 ± 0.71	46.5	65.10 ± 0.62	63.8
QSFormer	52.68 ± 0.65	50.9	70.45 ± 0.58	69.2
Askari et al.	55.14 ± 0.60	53.8	73.82 ± 0.51	72.5
AMPL	56.30 ± 0.58	55.2	75.05 ± 0.49	73.9
KLSANet	57.82 ± 0.63	56.9	75.94 ± 0.53	75.1
**HNeuroNet**	**62.15 ± 0.55**	**61.8**	**80.36 ± 0.45**	**79.5**

Reported results are Acc(%) with 95% confidence intervals (CI) and F1 scores (%). Bold data indicates optimal results.

To ensure a fair comparison with general few-shot learning methods, the evaluation on the Mini-ImageNet benchmark adhered to the standard intra-domain protocol, where the model was meta-trained exclusively on the 64 base classes of Mini-ImageNet and evaluated on its 20 novel classes. This intra-domain evaluation confirms the algorithmic universality of the DMP strategy beyond agricultural data. As shown in **Table 4**, under the 5-way 1-shot setting, HNeuroNet achieved an accuracy of 71.36%, surpassing the state-of-the-art meta-prompt learning method, AMPL (68.84%). This result indicates that the Dynamic Meta-Plasticity (DMP) strategy is not only applicable to disease recognition in specific agricultural domains but also effectively establishes context-dependent feature discriminative boundaries via the bi-level optimization system when facing general categories with large spans in Mini-ImageNet. This confirms that the simulated environmental homeostatic adaptation process of biological nervous systems possesses intrinsic robustness.

**Table 4:** Comparison of few-shot classification performance on the Mini-ImageNet dataset.

Method	5-way 1-shot		5-way 5-shot	
	Acc	F1	Acc	F1
Hiller et al.	63.45 ± 0.64	62.1	79.82 ± 0.51	78.9
QSFormer	65.78 ± 0.60	64.9	81.56 ± 0.48	80.6
KLSANet	66.82 ± 0.58	65.5	82.34 ± 0.46	81.4
Askari et al.	67.95 ± 0.55	66.8	83.10 ± 0.45	82.2
AMPL	68.84 ± 0.52	67.9	84.05 ± 0.42	83.3
**HNeuroNet**	**71.36 ± 0.49**	**70.5**	**85.68 ± 0.38**	**84.9**

Reported results are Acc(%) with 95% confidence intervals (CI) and F1 scores (%). Bold data indicates optimal results.

that transcends specific domains. Although QSFormer and AMPL leverage the advantages of Transformer long-range dependency modeling and prompt learning adaptability, respectively, HNeuroNet is capable of generating task-exclusive affine transformation parameters under single-sample conditions. This parameter generation mechanism reconstructs the feature space more directly than the prompt fine-tuning of AMPL, thereby establishing a performance gap of approximately 2.5% in the extreme few-shot (1-shot) scenario. In the 5-shot task, the 95% confidence interval of HNeuroNet narrowed to ±0.38%, and the F1 score reached 84.9%. This demonstrates that the model maintains extremely high statistical stability and accuracy even within general benchmarks characterized by highly diverse category distributions.

Furthermore, an analysis of the performance degradation from the 5-shot to the 1-shot scenario reveals the unique resilience of the proposed mechanisms under extreme data scarcity. While a natural accuracy drop is observed across all evaluated methods when reduced to a single support sample, HNeuroNet establishes the most robust discriminative boundaries. This sustained effectiveness is attributed to the NMG’s ability to achieve instantaneous weight reconstruction without extensive gradient iterations, overcoming the structural rigidity typical of conventional few-shot adaptations. Simultaneously, the HSM effectively filters background noise by relying on the temporal tracking of historical channel activity rather than large intra-class sample volumes, ensuring that high signal-to-noise ratio features are preserved even when prior knowledge is restricted to a single instance.

### 4.4 Visualization analysis of pest and disease images

**Figure 5:**
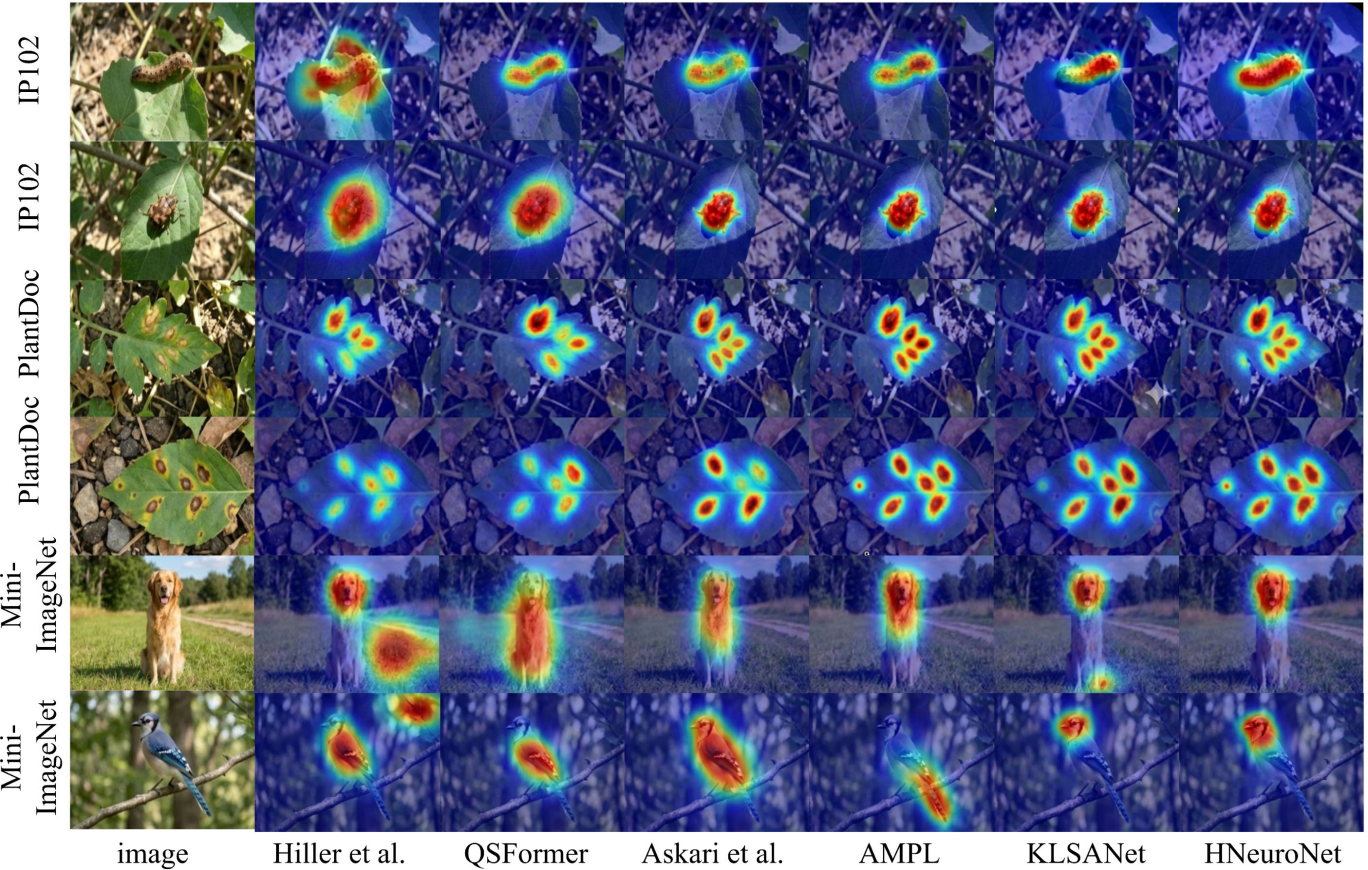
Visualization heatmaps of various few-shot recognition methods.

To provide an intuitive assessment of the model’s feature focusing capabilities and noise robustness within complex unstructured environments, **Figure 5** presents a comparison of Grad-CAM visualization heatmaps between H-NeuroNet and mainstream baseline methods, including QSFormer, AMPL, and KLSANet, on cross-domain samples. Observation of the visualization results reveals that in the complex field scenarios of the IP102 and PlantDoc agricultural datasets (such as the pest images affected by illumination in the first row and the complex soil backgrounds in the third row), the comparative methods exhibit varying degrees of attention diffusion. In particular, Transformer-based architectures, such as QSFormer and the method proposed by Hiller et al., lack dynamic filtering mechanisms for environmental noise. Consequently, their heatmaps frequently show erroneous activation in non-target regions (background leaves or weeds), resulting in a low signal-to-noise ratio for feature extraction. In contrast, H-NeuroNet demonstrates superior target localization precision and background suppression effects across all test samples. Whether in the fine-grained recognition of minute lesions or the detection of general objects in Mini-ImageNet, the regions of high activation (indicated in red) produced by the proposed method tightly cover the surface of the core target. Furthermore, the edge contours are distinct, with almost no background leakage. This advantage is significantly attributed to the HSM. By simulating the sliding threshold characteristics of BCM theory in the visual cortex, the model is capable of dynamically truncating high-frequency background signals based on historical statistical distributions. When combined with the instantaneous reconstruction of feature channels by the NMG, H-NeuroNet effectively overcomes the deficiency of feature discriminability inherent in traditional models under extreme few-shot conditions. This validates the superiority of the Dynamic Meta-Plasticity strategy in enhancing visual saliency and cross-domain generalization capabilities.

### 4.5 Cross-domain generalization analysis

To rigorously evaluate the true cross-domain adaptation capabilities of the proposed framework under severe domain shifts, we conducted an additional cross-domain experiment. Specifically, all models were meta-trained exclusively on the base classes of the general Mini-ImageNet dataset and subsequently meta-tested directly on the specialized agricultural datasets (IP102 and PlantDoc) without any target-domain fine-tuning.

Table legend: Reported results are 5-way 5-shot Acc (%). Bold data is the best results.

As detailed in **Table 5**, the massive domain shift—from general objects to unstructured field lesions and insects, causes a natural performance degradation across all evaluated models. However, HNeuroNet exhibits the most robust cross-domain generalization. For instance, when tested on the high-noise PlantDoc dataset (5-way 5-shot), HNeuroNet achieves an accuracy of 65.42%, outperforming the prompt-tuning AMPL method by 4.15%. This suggests that the NMG and HSM successfully decouple task-specific rapid adaptation and noise filtering from the rigid meta-trained backbone weights. Consequently, the model establishes robust discriminative boundaries even when prior knowledge is derived from an entirely disparate source domain, highlighting its immense potential for deployment in novel, data-scarce agricultural environments.

**Table 5:** Cross-domain few-shot classification performance (Meta-trained on Mini-ImageNet and Metatested on IP102 and PlantDoc).

Method	IP102	PlantDoc
Hiller et al.	54.20	49.35
QSFormer	58.15	54.10
Askari et al.	60.80	57.65
KLSANet	62.45	59.80
AMPL	63.10	61.27
HNeuroNet	**68.35**	**65.42**

The bolded values represent the best results.

## 5 Discussion

### 5.1 Hyperparameter analysis

**Figure 6:**
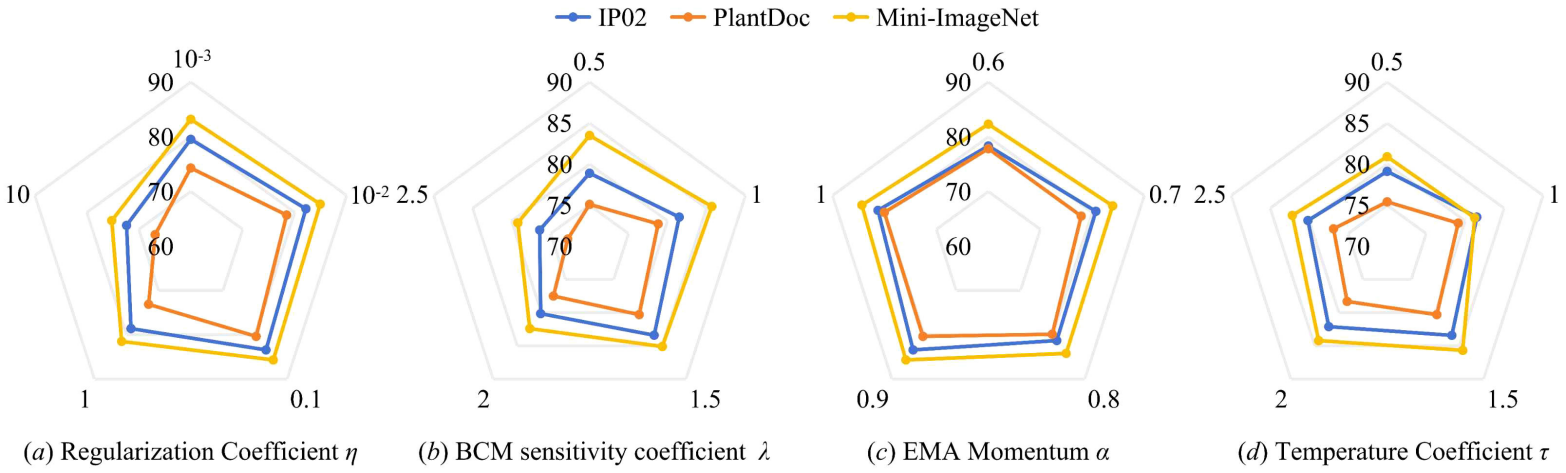
Radar chart of hyperparameter evaluation results for HNeuroNet.

HNeuroNet incorporates critical hyperparameters that govern its dynamic plasticity and homeostatic regulation. As illustrated in the comprehensive radar charts (**Figure 6**), we systematically evaluate the sensitivity of the model to four primary parameters: the homeostatic sparse regularization coefficient *η*, the BCM sensitivity coefficient *λ*, the EMA momentum *α*, and the temperature coefficient *τ*.

The coefficient *η* (**Figure 6a**) governs the weight of the sparse regularization term. Optimal performance centers around 0.1 across all datasets. A lower *η* fails to effectively drive the HSM to filter background noise, whereas an excessively high *η* compels excessive feature sparsity, leading to the loss of information regarding minute lesions. The coefficient λ (**Figure 6b**) regulates the dynamic response magnitude of the BCM sliding threshold. HNeuroNet maintains stable high performance within the interval λ ∈ [1.0,1.5]. However, under the extreme setting of λ = 2.5, performance on PlantDoc collapses significantly, suggesting that excessively high sensitivity thresholds are destructive to highly camouflaged disease features. The EMA momentum *α* (**Figure 6c**) controls the temporal tracking of historical channel activity. The results indicate that an optimal *α* near 0.9 effectively captures the normative background statistics. Lowering or raising this value heavily degrades performance, as it may cause the model to either overreact to sudden.

single-frame perturbations or fail to adapt to legitimate environmental shifts. The temperature coefficient *τ* (**Figure 6d**) scales the cosine similarity in the prototype metric space. Performance trends suggest that setting *τ* near 1.0 optimally balances intra-class compactness and inter-class separability, with accuracy dropping when the distribution becomes overly smoothed (*τ* ≤ 0.5) or excessively sharp (*τ* ≥ 2.0). Finally, secondary variables are theoretically constrained: the BCM non-linear exponent is fixed at *p* = 2 to simulate energy relationships, and the residual coefficient *β* acts as a minimal constant to ensure gradient stability, allowing the optimization focus to remain on the aforementioned primary dynamic coefficients.

### 5.2 Efficiency analysis

To comprehensively assess the deployment potential of the model within practical agricultural scenarios, this study evaluates the parameter efficiency, computational cost (FLOPs), and adaptation latency of the comparative methods, including the gradient-based baseline MAML. As detailed in **Table 6**, while Transformer-based architectures (QSFormer and Hiller et al.) leverage self-attention mechanisms to effectively capture long-range dependencies, their inherent computational complexity results in significant inference latency, rendering them ill-suited for real-time detection requirements. Similarly, methods such as KLSANet introduce pixel-level semantic alignment modules; while these enhance the precision of feature matching, they impose a substantial computational burden.

**Table 6:** Comparison of computational cost and adaptation latency across different few-shot learning methods.

Method	Adaptation mechanism	Params (M)	FLOPs (G)	Latency (ms/task)
MAML	Gradient Fine-tuning	11.2	112.5	1245.0
Hiller et al.	Metric Learning	12.6	55.2	89.5
QSFormer	Metric Learning	10.3	40.1	65.2
KLSANet	Metric Learning	9.6	29.5	45.8
Askari et al.	Metric Learning	6.8	34.2	38.4
AMPL	Prompt Fine-tuning	6.3	36.8	412.5
HNeuroNet (Ours)	Zero-gradient Generation	5.7	35.6	14.2

To empirically validate the efficiency advantage of the zero-gradient parameter reconstruction, we compared the adaptation phase cost against MAML and the prompt-tuning AMPL. Methods relying on iterative backpropagation, such as MAML, incur massive adaptation latency (1245.0 ms) and computational overhead (112.5 GFLOPs) due to inner-loop gradient steps. While AMPL utilizes prompt fine-tuning, it still requires gradient updates during adaptation, leading to considerable latency. In contrast, HNeuroNet exhibits a decisive advantage. The NMG innovatively employs a lightweight hypernetwork architecture capable of instantaneously generating task-adaptive affine parameters based on the support set. This parameter reconstruction mechanism completely circumvents the high computational costs associated with test-time gradient iterations, achieving rapid adaptation. Furthermore, the HSM involves only low-overhead statistical calculations and threshold truncation, introducing negligible additional floating-point operations. In summary, HNeuroNet achieves dual optimization of classification accuracy and inference speed with only a marginal increase in parameters, demonstrating its high application value for deployment on resource-constrained agricultural edge computing devices.

### 5.3 Controlled noise robustness experiment

To precisely isolate and quantify the contribution of the HSM to noise invariance, we designed a controlled robustness experiment utilizing a subset of the Mini-ImageNet dataset (5-way 5-shot). Following standard perturbation protocols, we introduced three distinct types of synthetic noise to the query set images: Gaussian blur (kernel size 5 × 5, *σ* = 2), random occlusion (20% of the image area masked), and extreme illumination changes (brightness factor adjusted randomly by ±50%). We evaluated all comparative methods alongside our proposed baseline and modules to systematically measure noise suppression capabilities. As detailed in **Table 7**, existing state-of-the-art methods and the baseline ResNet-12 model experience severe performance degradation when exposed to these synthetic interferences. For instance, while methods like AMPL and KLSANet achieve high accuracy on clean images, their performance drops significantly under partial occlusion and drastic illumination shifts. In contrast, the integration of the HSM module (Baseline + HSM) substantially mitigates this degradation. Under extreme illumination changes the Baseline + HSM maintains an accuracy of 75.82%, outperforming the standard baseline by 6.45%. The complete HNeuroNet framework further secures the most robust discriminative boundaries across all noise types. This controlled evaluation suggests that the dynamic sliding threshold mechanism, which adaptively regulates neuron activation based on historical feature statistics, is highly effective at filtering structured visual perturbations, thereby preserving feature purity and stability.

**Table 7:** Performance comparison Acc(%) of various methods under controlled synthetic noise on the Mini-ImageNet dataset (5-way 5-shot).

Method	Clean images	Gaussian blur	Occlusion (20%)	Illumination
Hiller et al.	79.82	73.15	68.50	71.80
QSFormer	81.56	75.20	71.30	74.60
KLSANet	82.34	78.50	73.60	76.10
Askari et al.	83.10	77.40	72.10	76.80
AMPL	84.05	78.80	74.50	77.20
Baseline	76.45	71.20	65.40	69.37
Baseline + HSM	78.92	76.15	72.80	75.82
HNeuroNet	85.68	83.40	80.15	82.50

### 5.4 Ablation experiment

To conduct an in-depth investigation into the contributions of the core components within the HNeuroNet framework, we performed an ablation analysis on three public datasets. The ResNet-12 architecture was initially selected as the primary baseline because its lightweight structure provides an optimal balance between computational efficiency and representative capacity, serving as a standard benchmark in few-shot learning scenarios. To rigorously verify that the proposed mechanisms are architecture-agnostic, we extended our ablation study to include the deeper ResNet-18 backbone. As presented in the updated **Table 8**, the experimental results compellingly validate the independent value and synergistic effects of the NMG, HSM, and DMP in addressing distinct challenges across both backbones. First, the solitary introduction of the NMG module significantly enhanced model performance on the IP102 and Mini-ImageNet datasets. Second, the HSM module demonstrated an irreplaceable role on the PlantDoc dataset, delivering a substantial leap in accuracy by effectively stripping away high-frequency background noise. Finally, the complete HNeuroNet, integrating the DMP strategy, achieved optimal performance across all tasks for both ResNet-12 and ResNet-18 architectures. This suggests that the dynamic parameter reconstruction and homeostatic background suppression introduced by our framework provide universal functional benefits, ensuring the gains are not specific to a single backbone.

**Table 8:** Ablation results of HNeuroNet components across different backbones (5-way 5-shot accuracy %).

Backbone	NMG	HSM	DMP	IP102	PlantDoc	Mini-ImageNet
ResNet-12	×	×	×	74.15	69.82	76.45
ResNet-12	✓	×	×	78.62	72.54	81.30
ResNet-12	×	✓	×	77.80	76.15	78.92
ResNet-12	✓	✓	×	81.58	78.90	83.45
ResNet-12	✓	✓	✓	83.42	80.36	85.68
ResNet-18	×	×	×	75.30	71.10	77.80
ResNet-18	✓	×	×	79.60	73.50	82.50
ResNet-18	×	✓	×	78.90	77.30	80.10
ResNet-18	✓	✓	×	82.70	80.10	84.60
ResNet-18	✓	✓	✓	**84.55**	**81.45**	**86.8**

Bold data indicates the best results.

To further quantify the unique computational contribution of the BCM-inspired HSM beyond increased model complexity, we conducted an additional comparative analysis pitting the HSM against established noise suppression techniques. Specifically, we evaluated the baseline ResNet-12 model equipped with Adaptive Batch Normalization (AdaBN) and a standard spatial attention module (Convolutional Block Attention Module, CBAM). As shown in **Table 9**, while AdaBN and CBAM offer marginal improvements in target localization, they remain sensitive to high-frequency agricultural noise and struggle to suppress unstructured backgrounds effectively. In contrast, the HSM’s dynamic sliding threshold mechanism, which regulates neuron response thresholds according to historical statistical distributions, consistently yields superior accuracy, particularly on the highly noisy PlantDoc dataset. This indicates that the biologically-plausible dynamic gating provided by HSM is computationally necessary for establishing robust discriminative boundaries under extreme data scarcity and complex field interference.

**Table 9:** Comparative analysis of HSM against established noise suppression techniques (5-way 5-shot accuracy %).

Method	IP102	PlantDoc	Mini-ImageNet
Baseline (ResNet-12)	74.15	69.82	76.45
Baseline + AdaBN	75.20	70.45	77.10
Baseline + CBAM	76.10	71.85	77.55
Baseline + HSM (Ours)	**77.80**	**76.15**	**78.92**

Bold data indicates the best results.

## 6 Conclusion

Targeting the dual challenges of extreme scarcity of emerging disease samples and unstructured background noise interference in precision agriculture, we proposes the bio-inspired HNeuroNet. This framework breaks the data-dependency paradigm of traditional static convolutional networks by simulating the synaptic homeostasis and neuromodulation mechanisms of the biological brain, endowing machine vision systems.

with dynamic plasticity and environmental adaptability under extreme few-shot conditions. First, the NMG utilizes a hypernetwork to achieve zero-gradient parameter reconstruction for new tasks, effectively overcoming structural rigidity and catastrophic forgetting in rapid adaptation inherent in traditional meta-learning. Second, the HSM introduces an adaptive sliding threshold based on BCM theory, successfully functioning as a dynamic high-pass filter to precisely strip away background artifacts caused by illumination and texture in low signal-to-noise ratio environments. Finally, the DMP couples parameter generation and threshold regulation within a bi-level optimization system, establishing a context-dependent feature interaction mode. Experimental results confirm that HNeuroNet achieves significantly superior performance on IP102, PlantDoc, and cross-domain benchmarks, particularly in the highly challenging 1-shot scenario, substantially enhancing the robustness of disease recognition.

However, the model’s performance depends to some extent on the quality of the support set samples. Specifically, in real-world agricultural scouting, support sets may frequently contain noisy, mislabeled, or heavily occluded examples. Because the NMG relies on global feature aggregation to construct the task context vector, the presence of mislabeled or highly anomalous samples may skew this statistical mean. Such deviations can subsequently lead to the generation of suboptimal affine modulation parameters, potentially causing the model to incorrectly calibrate channel gains and prioritize irrelevant artifacts over core lesion features. This failure mode suggests that while the NMG enables rapid adaptation, its robustness to imperfect support sets may be compromised under extreme noise. Future work will integrate robust prototype estimation algorithms and multi-modal data, alongside self-supervised pre-training techniques, to further reduce reliance on pristine manually annotated samples, thereby constructing agricultural agents with stronger environmental perception and universality.

## Data Availability

The original contributions presented in the study are included in the article/supplementary material. Further inquiries can be directed to the corresponding author.

## References

[B1] Ali M. A. Sharma A. K. Dhanaraj R. K. (2024). Heterogeneous features and deep learning networks fusion-based pest detection, prevention and controlling system using IoT and pest sound analytics in a vast agriculture system. Comput. Electr. Eng. 116, 109146. doi: 10.1016/j.compeleceng.2024.109146. PMID: 41853590

[B2] Asadi-Aghbolaghi M. Darbandsari A. Zhang A. Contreras-Sanz A. Boschman J. Ahmadvand P. . (2024). Learning generalizable AI models for multi-center histopathology image classification. Np Precis. Onc. 8, 151. doi: 10.1038/s41698-024-00652-4. PMID: 39030380 PMC11271637

[B3] Askari F. Fateh A. Mohammadi M. R. (2025). Enhancing few-shot image classification through learnable multi-scale embedding and attention mechanisms. Neural Networks 187, 107339. doi: 10.1016/j.neunet.2025.107339. PMID: 40090300

[B4] Banfi D. Bianchi T. Mastore M. Brivio M. F. (2024). Optimization of experimental infection of the animal model Galleria mellonella Linnaeus 1758 (Lepidoptera: Pyralidae) with the Gram-Positive bacterium Micrococcus luteus. Insects 15, 618. doi: 10.3390/insects15080618. PMID: 39194822 PMC11354611

[B5] Bashir R. N. Khan F. A. Khan A. A. Tausif M. Abbas M. Z. Shahid M. M. A. . (2023). Intelligent optimization of reference evapotranspiration (ETo) for precision irrigation. J. Comput. Sci. 69, 102025. doi: 10.1016/j.jocs.2023.102025. PMID: 41853590

[B6] Cao Z. Xu G. Gao Y. Xu J. Tian F. Shi H. . (2025). Development, deployment, and feature interpretability of a three-class prediction model for pulmonary diseases. Insights Imaging 16, 133. doi: 10.1186/s13244-025-02020-7. PMID: 40571839 PMC12202249

[B7] Chodey M. D. Noorullah Shariff C. (2023). Pest detection via hybrid classification model with fuzzy C-means segmentation and proposed texture feature. Biomed. Signal Process. Control 84, 104710. doi: 10.1016/j.bspc.2023.104710. PMID: 41853590

[B8] Dai G. Tian Z. Fan J. Sunil C. Dewi C. (2024). DFN-PSAN: Multi-level deep information feature fusion extraction network for interpretable plant disease classification. Comput. Electron. Agric. 216, 108481. doi: 10.1016/j.compag.2023.108481. PMID: 41853590

[B9] Dhollander S. Balmos O.-M. Cattaneo E. Cortiñas J. A. Boklund A. E. Szczotka-Bochniarz A. . (2025). Investigating the role of stable flies (Stomoxys calcitrans) and biting midges of the genus Culicoides as potential mechanical vectors of African swine fever virus in Lithuania, Poland and Romania. Parasites Vectors 18, 312. doi: 10.1186/s13071-025-06816-w. PMID: 40745323 PMC12312455

[B10] Dong Z. (2025). Few-shot fast adaptation strategies with meta-learning and multi-armed bandits. ACE 183, 59–66. doi: 10.54254/2755-2721/2025.BJ26897. PMID: 35591207

[B11] Fu Y. Xiang L. Zahid Y. Ding G. Mei T. Shen Q. . (2022). Long-tailed visual recognition with deep models: A methodological survey and evaluation. Neurocomputing 509, 290–309. doi: 10.1016/j.neucom.2022.08.031. PMID: 41853590

[B12] Ge P. Lu X. Zhao W. Wu S. Smith N. Gao L. . (2025). Application of a dual-loop double-stranded RNA to control small brown planthopper. Insect Sci., 1744–7917, 70128. doi: 10.1111/1744-7917.70128. PMID: 40654022

[B13] Hiller M. Ma R. Harandi M. Drummond T. (2022). “ Rethinking generalization in few-shot classification,” in 36th conference on neural information processing systems, neurips 2022, november 28, 2022 - december 9, 2022, vol. 35. (New Orleans, USA: Neural information processing systems foundation), 1–14.

[B14] Jacoba C. M. P. Doan D. Salongcay R. P. Aquino L. A. C. Silva J. P. Y. Salva C. M. G. . (2023). Performance of automated machine learning for diabetic retinopathy image classification from multi-field handheld retinal images. Ophthalmol. Retina 7, 703–712. doi: 10.1016/j.oret.2023.03.003. PMID: 36924893

[B15] Jhaiaun P. Rudeekiatthamrong A. Chimnoi W. Nguyen G. T. Ngasaman R. Phasuk J. . (2025). Molecular detection of hemoparasites in hematophagous insects collected from livestock farms in Northeastern Thailand. Insects 16, 207. doi: 10.3390/insects16020207. PMID: 40003837 PMC11856380

[B16] Khan M. A. Akram T. Sharif M. Awais M. Javed K. Ali H. . (2018). CCDF: Automatic system for segmentation and recognition of fruit crops diseases based on correlation coefficient and deep CNN features. Comput. Electron. Agric. 155, 220–236. doi: 10.1016/j.compag.2018.10.013. PMID: 41853590

[B17] Khan M. A. Akram T. Sharif M. Saba T. (2020). Fruits diseases classification: Exploiting a hierarchical framework for deep features fusion and selection. Multimedia Tools Appl. 79, 25763–25783. doi: 10.1007/s11042-020-09244-3. PMID: 41853694

[B18] Khan A. A. Faheem M. Bashir R. N. Wechtaisong C. Abbas M. Z. (2022a). Internet of things (IoT) assisted context aware fertilizer recommendation. IEEE Access 10, 129505–129519. doi: 10.1109/ACCESS.2022.3228160. PMID: 41116384

[B19] Khan A. A. Nauman M. A. Bashir R. N. Jahangir R. Alroobaea R. Binmahfoudh A. . (2022b). Context aware evapotranspiration (ETs) for saline soils reclamation. IEEE Access 10, 110050–110063. doi: 10.1109/ACCESS.2022.3206009. PMID: 41116384

[B20] Kolivand H. Fern B. M. Saba T. Rahim M. S. M. Rehman A. (2019). A new leaf venation detection technique for plant species classification. Arabian J. Sci. Eng. 44, 3315–3327. doi: 10.1007/s13369-018-3504-8. PMID: 41853694

[B21] Li D. Zhang C. Li J. Li M. Huang M. Tang Y. (2024). MCCM: Multi-scale feature extraction network for disease classification and recognition of chili leaves. Front. Plant Sci. 15. doi: 10.3389/fpls.2024.1367738. PMID: 38863551 PMC11165206

[B22] Liu Y. Mazumdar S. Bath P. A. (2023). An unsupervised learning approach to diagnosing 888 Alzheimer’s disease using brain magnetic resonance imaging scans. Int. J. Med. Inf. 173, 105027. doi: 10.1016/j.ijmedinf.2023.105027. PMID: 36921480

[B23] Loesing H. Bartelt S. Cvjetkovic V. Soeckler-Lionetti C. Bechmann L. Kipschull K. . (2025). Assessment of sponge sampling for real-time PCR detection of Cystoisospora suis from environmental and faecal samples from piglet-producing farms. Porc Health Manag 11, 43. doi: 10.1186/s40813-025-00454-5. PMID: 40745352 PMC12315398

[B24] Mukhtar H. Khan M. Z. Usman Ghani Khan M. Saba T. Latif R. (2021). “ Wheat plant counting using UAV images based on semi-supervised semantic segmentation,” in 2021 1st international conference on artificial intelligence and data analytics (CAIDA). (Riyadh, Saudi Arabia: IEEE), 257–261. doi: 10.1109/CAIDA51941.2021.9425252, PMID:

[B25] Nakayama Y. Sato M. Okamoto M. Kondo Y. Tamura M. Minagawa Y. . (2023). Deep learning-based classification of adequate sonographic images for self-diagnosing deep vein thrombosis. PloS One 18, e0282747. doi: 10.1371/journal.pone.0282747. PMID: 36877716 PMC9987812

[B26] Nandhini C. Brindha M. (2024). Visual regenerative fusion network for pest recognition. Neural Comput. Applic. 36, 2867–2882. doi: 10.1007/s00521-023-09173-w. PMID: 41853694

[B27] Naqi S. A. E. A. Iqbal K. Khan A. A. Khan R. Jamil S. Ishtiaq U. (2025). Diseases detection from Apple leaf using deep transfer learning approach. Int. J. Theor. Appl. Comput. Intell., 2025, 70. doi: 10.65278/IJTACI.2025.13

[B28] Omara J. Talavera E. Otim D. Turcza D. Ofumbi E. Owomugisha G. (2023). A field-based recommender system for crop disease detection using machine learning. Front. Artif. Intell. 6. doi: 10.3389/frai.2023.1010804. PMID: 37181731 PMC10171456

[B29] Parashar N. Johri P. Khan A. Gaur N. Kadry S. (2024). An integrated analysis of yield prediction models: A comprehensive review of advancements and challenges. Computers Materials Continua 80, 389–425. doi: 10.32604/cmc.2024.050240. PMID: 40612875

[B30] Pourpanah F. Abdar M. Luo Y. Zhou X. Wang R. Lim C. P. . (2022). A review of generalized zero-shot learning methods. IEEE Trans. Pattern Anal. Mach. Intell., 1, 20. doi: 10.1109/TPAMI.2022.3191696. PMID: 35849673

[B31] Ragnarsdottir H. Ozkan E. Michel H. Chin-Cheong K. Manduchi L. Wellmann S. . (2024). Deep learning based prediction of pulmonary hypertension in newborns using echocardiograms. Int. J. Comput. Vis. 132, 2567–2584. doi: 10.1007/s11263-024-01996-x. PMID: 38911323 PMC11186939

[B32] Sajitha P. Andrushia A. D. Anand N. Naser M. (2024). A review on machine learning and deep learning image-based plant disease classification for industrial farming systems. J. Ind. Inf. Integr. 38, 100572. doi: 10.1016/j.jii.2024.100572. PMID: 41853590

[B33] Sanaeifar A. Guindo M. L. Bakhshipour A. Fazayeli H. Li X. Yang C. (2023). Advancing precision agriculture: The potential of deep learning for cereal plant head detection. Comput. Electron. Agric. 209, 107875. doi: 10.1016/j.compag.2023.107875. PMID: 41853590

[B34] Sharma V. Tripathi A. K. Mittal H. (2022). Technological revolutions in smart farming: Current trends, challenges & future directions. Comput. Electron. Agric. 201, 107217. doi: 10.1016/j.compag.2022.107217. PMID: 41853590

[B35] Singh S. P. Dhiman G. Juneja S. Viriyasitavat W. Singal G. Kumar N. . (2024). A new QoS optimization in IoT-smart agriculture using rapid-adaption-based nature-inspired approach. IEEE Internet Things J. 11, 5417–5426. doi: 10.1109/JIOT.2023.3306353. PMID: 41116384

[B36] Singh D. Jain N. Jain P. Kayal P. Kumawat S. Batra N. (2020). “ PlantDoc: A dataset for visual plant disease detection,” in CoDS COMAD 2020. 249–253 (New Orleans, USA: Association for Computing Machinery). doi: 10.1145/3371158.3371196

[B37] Skaldina O. Łukowski A. Leskinen J. T. Koistinen A. P. Eeva T. (2023). Mobile samplers of particulate matter – Flying omnivorous insects in detection of industrial contamination. Sci. Total Environ. 867, 161511. doi: 10.1016/j.scitotenv.2023.161511. PMID: 36632898

[B38] Sun Z. Zheng W. Guo P. (2024). KLSANet: Key local semantic alignment network for few-shot image classification. Neural Networks 178, 106456. doi: 10.1016/j.neunet.2024.106456. PMID: 38901096

[B39] V. P. Kumar A. M. S. Praveen J. I. R. Venkatraman S. Kumar S. P. Aravintakshan S. A. . (2024). Improved tomato leaf disease classification through adaptive ensemble models with exponential moving average fusion and enhanced weighted gradient optimization. Front. Plant Sci. 15. doi: 10.3389/fpls.2024.1382416. PMID: 38828218 PMC11140105

[B40] Vinyals O. Blundell C. Lillicrap T. Kavukcuoglu K. Wierstra D. (2016). “ Matching networks for one shot learning,” in NIPS’16. 3637–3645 (New Orleans, USA: Curran Associates Inc).

[B41] Vishnoi V. K. Kumar K. Kumar B. Mohan S. Khan A. A. (2023). Detection of Apple plant diseases using leaf images through convolutional neural network. IEEE Access 11, 6594–6609. doi: 10.1109/ACCESS.2022.3232917. PMID: 41116384

[B42] Waikel R. L. Othman A. A. Patel T. Ledgister Hanchard S. Hu P. Tekendo-Ngongang C. . (2024). Recognition of genetic conditions after learning with images created using generative artificial intelligence. JAMA Netw. Open 7, e242609. doi: 10.1001/jamanetworkopen.2024.2609. PMID: 38488790 PMC10943405

[B43] Wang X. Wang X. Jiang B. Luo B. (2023). Few-shot learning meets transformer: Unified query-support transformers for few-shot classification. IEEE Trans. Circuits Syst. Video Technol. 33, 7789–7802. doi: 10.1109/TCSVT.2023.3282777. PMID: 41116384

[B44] Watson G. Taylor J. Lambert W. T. Beavers K. Kirk D. Walsh M. J. . (2025). Behavioral profiling in zebrafish identifies insecticide-related compounds. J. Agric. Food. Chem. 73, 2805–2813. doi: 10.1021/acs.jafc.4c09342. PMID: 39854692 PMC11803735

[B45] Wu Z. Huai L. Liu T. Shangguan Z. Wang L. Huo J. . (2025). AMPL: An adaptive meta-prompt learner for few-shot image classification. Neural Networks, 108288. doi: 10.1016/j.neunet.2025.108288. PMID: 41242073

[B46] Wu X. Zhan C. Lai Y.-K. Cheng M.-M. Yang J. (2019). “ IP102: A large-scale benchmark dataset for insect pest recognition,” in 2019 IEEE/CVF conference on computer vision and pattern recognition (CVPR). (Riyadh, Saudi Arabia: IEEE), 8779–8788. doi: 10.1109/CVPR.2019.00899, PMID:

[B47] Zheng Z. Zhang C. (2022). Electronic noses based on metal oxide semiconductor sensors for detecting crop diseases and insect pests. Comput. Electron. Agric. 197, 106988. doi: 10.1016/j.compag.2022.106988. PMID: 41853590

[B48] Zhou Y. Zhang H. Liu D. Khashaveh A. Li Q. Wyckhuys K. A. G. . (2023). Long-term 966 insect censuses capture progressive loss of ecosystem functioning in East Asia. Sci. Adv. 9, eade9341. doi: 10.1126/sciadv.ade9341. PMID: 36735783 PMC9897670

